# Spatially informed reference-free cell-type deconvolution for spatial transcriptomics with SpatialCD

**DOI:** 10.1101/gr.281829.125

**Published:** 2026-07

**Authors:** Phuong Vo, Yuehua Cui

**Affiliations:** Department of Statistics and Probability, Michigan State University, East Lansing, Michigan 48824, USA

## Abstract

Cell-type deconvolution has been instrumental for the analysis of spatial transcriptomics (ST) data to reveal underlying tissue heterogeneity. Although reference-based methods have been widely explored, practical limitations, particularly the need for matched single-cell RNA-seq data sets, highlight the value of robust reference-free methods. Existing reference-free approaches, such as STdeconvolve, overlook spatial information, despite the well-established observation that spatially adjacent spots often share similar cellular compositions. Motivated by this, we propose SpatialCD, a spatially informed reference-free deconvolution method that extends Latent Dirichlet Allocation (LDA) with spatial regularization to encourage neighboring spots to exhibit similar cell-type structures. SpatialCD produces improved estimates of cell-type proportions and gene expression profiles. Across simulated and real data sets, including MERFISH-derived simulations, mouse olfactory bulb (MOB), 10× Visium, and DBiT-seq data, SpatialCD consistently improves performance over existing reference-free methods across evaluated data sets by recovering more accurate transcriptional patterns and revealing biologically coherent spatial organization across normal and diseased tissues, including subtle anatomical layers and region-specific tumor-associated cell populations. This work advances statistical tools for spatial transcriptomics and enriches the methodological toolkit for complex spatial gene expression analysis.

Understanding the spatial organization of transcriptionally distinct cell types within tissues is essential to uncover tissue structure and function (Ståhl et al. 2016; Zhang et al. 2025). The advent of spatial transcriptomics (ST) has significantly enhanced our ability to analyze gene expression in situ, enabling detailed mapping of cell-type distribution and gene expression patterns at multicellular resolution. Over time, various cell-type deconvolution algorithms have been developed specifically for analyzing ST data from spatial barcoding-based technologies such as 10× Visium (Stickels et al. 2021), DBiT-seq (Liu et al. 2020), and Slide-seq (Rodriques et al. 2019). A recent review broadly categorizes spatial deconvolution methods into reference-based and reference-free approaches (Gaspard-Boulinc et al. 2025). Reference-based methods leverage annotated single-cell RNA-seq (scRNA-seq) data to model each spatial spot as a mixture of predefined cell-type expression profiles. These approaches benefit from external single-cell references and can align results with established biological knowledge. In contrast, reference-free methods infer latent transcriptional programs directly from ST data, without relying on predefined cell-type signatures, and typically incorporate prior knowledge only at the annotation stage.

Despite the growing availability of scRNA-seq data, reference-based approaches depend critically on the availability of high-quality, well-matched reference data. In practice, such references could be incomplete or mismatched to the target tissue. Differences in tissue source, disease state, developmental stage, or experimental protocol can introduce substantial batch effects and systematic biases between scRNA-seq and ST data, which may compromise deconvolution accuracy. In addition, scRNA-seq data may fail to capture rare cell populations, transitional states, or fine-grained subtypes, particularly in complex or diseased tissues. Because reference-based methods are restricted to the cell types present in the reference panel, they are inherently limited in their ability to detect novel or previously uncharacterized cellular states. In contrast, reference-free approaches infer latent transcriptional programs directly from the observed gene expression data, enabling the identification of context-specific cell populations when reference data lack sufficient resolution or coverage. This flexibility is particularly important for emerging spatial technologies such as MERFISH and Visium HD, which may use targeted gene panels or operate at resolutions that differ substantially from available scRNA-seq data. Differences in gene coverage and measurement platforms further complicate direct transfer of reference profiles. As a result, reference-free deconvolution remains essential for accurately characterizing tissue organization and disease-associated transcriptional programs across diverse experimental settings. This need has motivated growing interest in developing reference-free deconvolution methods.

Among current reference-free approaches, STdeconvolve (Miller et al. 2022) and SpiceMix (Chidester et al. 2023) are some of the early developed methods for this purpose. STdeconvolve introduces a Latent Dirichlet Allocation (LDA) (Blei et al. 2003) framework to infer cell-type composition and transcriptional profiles directly from ST data. However, a major drawback of STdeconvolve is its failure to incorporate spatial locations of the spots from which gene expression data are collected, despite evidence that spatially adjacent spots often share similar cell-type compositions. SpiceMix builds upon the Non-Negative matrix factorization (NMF) model, incorporating spatial graph information by applying hidden Markov random fields (HMRF) to deconvolve each spot as a mixture of latent factors (metagenes). However, it is not specifically tailored for spatial cell-type deconvolution (Liu et al. 2023b), and lacks a built-in criterion for selecting the optimal number of deconvolved cell types (Chidester et al. 2023). As a result, these approaches may fail to fully capture spatial organization or accurately distinguish cell-type patterns in real data sets.

In this study, we aim to address these limitations by introducing SpatialCD, a Spatially informed, reference-free Cell type Deconvolution framework for multicellular ST data. SpatialCD extends the LDA model (Blei et al. 2003) by incorporating graph-based spatial regularization that encourages smoothness in cell-type composition across neighboring spots, overcoming the independence assumption of STdeconvolve. Specifically, our approach uses the spatial coordinates of each spot to identify its *k*-nearest neighbors, and then a regularized deconvolution problem is solved using the Alternating Direction Method of Multipliers (ADMM) (Boyd et al. 2011). The method also provides a data-driven approach for selecting the number of deconvolved cell types and prioritizes overdispersed, spatially informative genes to enhance robustness. We evaluate SpatialCD on both simulated and real ST data sets generated from various spatial transcriptomics technologies, including MERFISH and ST platforms.

## Results

### Method overview

**Figure 1.**
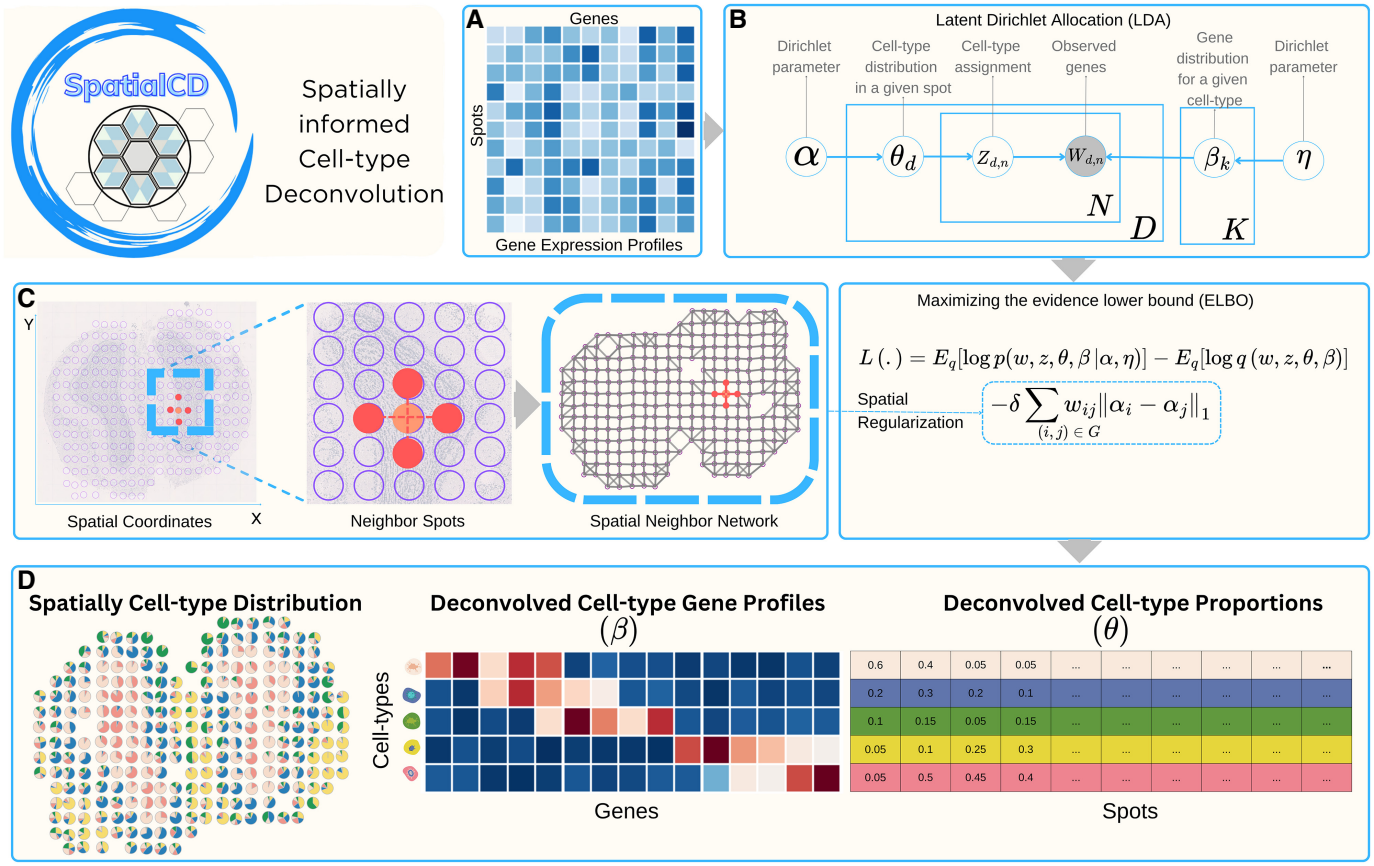
Overview of SpatialCD. (*A*) SpatialCD takes as input spatial transcriptomics data represented by a gene expression matrix (spot-by-gene matrix) and the corresponding spatial coordinates of spots (spot-by-2 matrix). (*B*) Graphical model of SpatialCD based on Latent Dirichlet Allocation (LDA), in which shaded nodes denote observed variables and unshaded nodes denote latent variables. (*C*) SpatialCD constructs a spatial nearest-neighbor graph and incorporates it into the LDA framework through spatial regularization terms. (*D*) SpatialCD simultaneously infers cell-type composition (*θ*, spot-by-cell-type matrix) and transcriptional profiles (*β*, cell-type-by-gene matrix). The proportions of deconvolved cell-types can be spatially visualized across the spots.

As illustrated in the method overview in Figure 1A–D, SpatialCD first constructs a nearest neighbor graph, in which each spot is a node in the graph with edges to its nearest neighbors. Next, SpatialCD implements a Latent Dirichlet Allocation (LDA) model (Blei et al. 2003) with spatial constraints to deconvolve the latent cell types with multi-cellular ST data. In our method, each spot is treated as a “document,” composed of a mixture of cell types, which serves as the latent topics uncovered by the LDA model (Blei et al. 2003). Each topic is characterized by a certain distribution of genes, which are considered as the “words” of the document. The LDA outputs contain the deconvolved proportions of cell types in each spot (*θ*), and the gene expression profiles for each cell type (*β*).

### SpatialCD accurately recovers cell-type composition and transcriptional profiles in simulated ST data

#### Mouse medial preoptic area (MPOA) data

**Figure 2.**
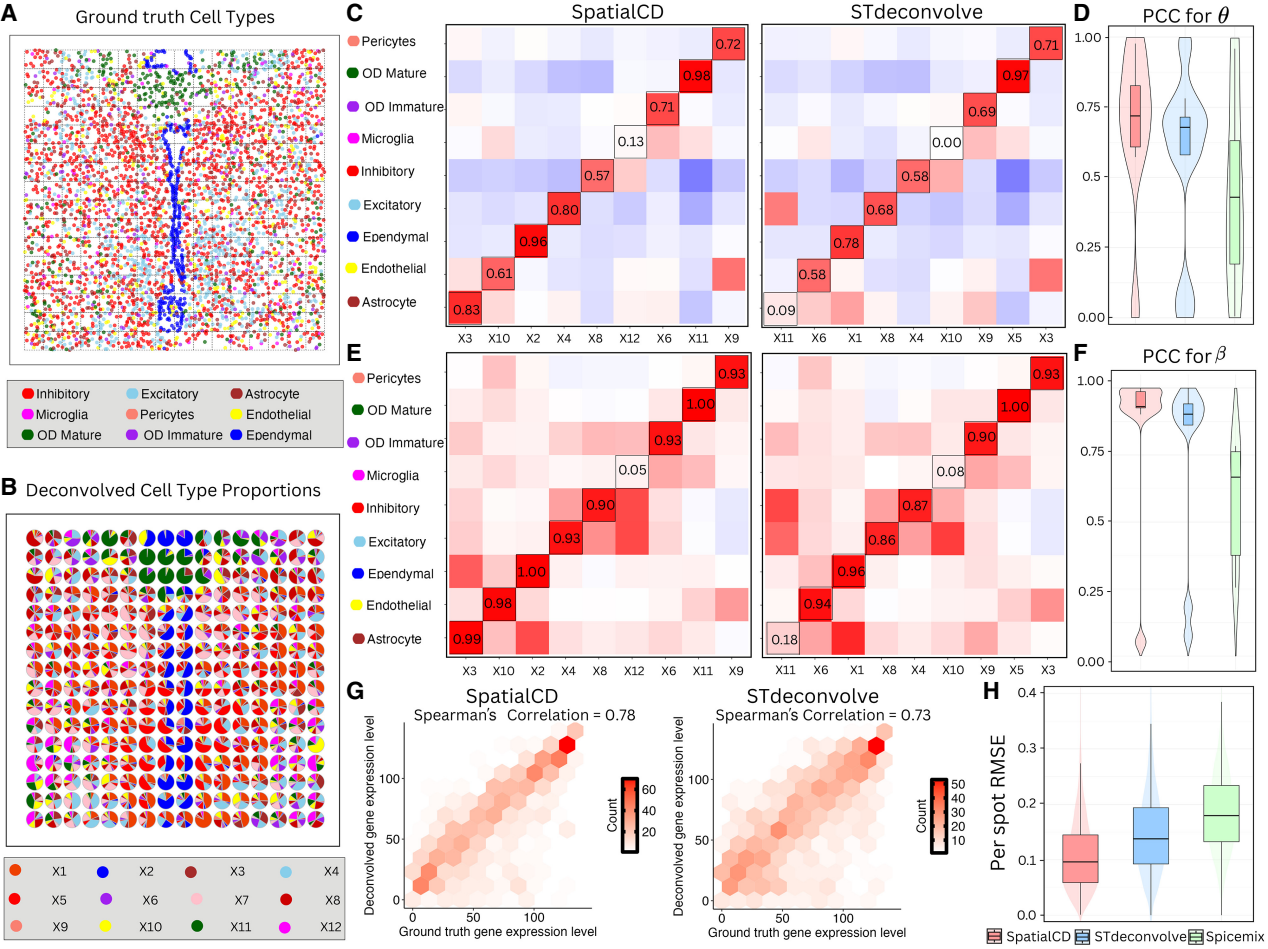
Deconvolution of simulated ST data (MPOA). (*A*) Ground truth single-cell resolution MERFISH data of one section of the MPOA, partitioned into 100 μm^2^ grids (gray squares). Each dot is a single cell colored by its ground truth cell-type label. (*B*) Proportions of deconvolved cell types from SpatialCD represented as pie charts for each simulated spot. (*C*) Comparison of cell-type composition (*θ*): Pearson’s correlation heatmaps between deconvolved and ground truth proportions using SpatialCD and STdeconvolve, with matched deconvolved and ground truth cell types highlighted with bounding boxes. (*D*) PCC for *θ*: Pearson’s correlation coefficients between the deconvolved cell-type composition and matched ground truth across spots for SpatialCD, STdeconvolve, and Spicemix. (*E*) Comparison of transcriptional profile (*β*): Pearson’s correlation heatmaps between deconvolved and ground truth gene expression profiles using SpatialCD and STdeconvolve, with matched deconvolved and ground truth cell types highlighted. (*F*) PCC for *β*: Pearson’s correlation coefficients between the deconvolved transcriptional profiles and matched ground truth profiles across genes for SpatialCD, STdeconvolve, and Spicemix. (*G*) Gene ranking based on expression levels in deconvolved transcriptional profiles compared to their rankings in the matched ground truth, along with the corresponding Spearman's correlation. (*H*) Boxplot of the root-mean-square error (RMSE) per spot of the deconvolved cell-type proportions compared to ground truth for SpatialCD, STdeconvolve, and Spicemix.

We first applied SpatialCD to a simulated ST data set generated from single-cell ST data of the mouse medial preoptic area (MPOA) (Chen et al. 2015; Moffitt et al. 2018), to evaluate the performance in recovering both cell-type compositions and transcriptional profiles. The MPOA data set includes 12 brain sections from different anterior-posterior positions of a single female mouse, with each section capturing spatial gene expression of 135 selected genes distinguishing 9 major cell types (Fig. 2A). Following the simulation framework of Miller et al. (2022), we generated multicellular ST data from single-cell MERFISH measurements of the mouse MPOA. After preprocessing and filtering, discrete transcript counts for 135 genes were retained for 59,651 cells annotated into 9 major cell types, excluding cells labeled as ambiguous. To simulate spot-level ST data, we aggregated single cells into square spatial pixels of 100 μm^2^ based on their spatial coordinates (Fig. 2A). For each tissue section, transcript counts of cells whose centroids fell within each grid square were summed to produce spot-level gene counts, and boundary pixels extending beyond the tissue region were discarded. This resulted in a simulated data set of 3072 spots × 135 genes. Ground-truth cell-type proportions were computed directly from the annotated cells contributing to each spot, and ground-truth transcriptional profiles were defined by averaging gene counts across cells of the same type and normalizing each profile to sum to one.

SpatialCD was then applied to this “simulated” data set to simultaneously infer the cell-type composition and transcriptional profile across all 12 sections. The model automatically identified the optimal number of deconvolved cell types as *K* = 12 based on the perplexity plot, which is the same as what STdeconvolve chooses (Supplemental Figs. S1A,B). When compared to SpiceMix (Chidester et al. 2023), we fixed the number of cell types as *K* = 9, equal to the number of cell types in the ground truth, because it does not have a mechanism to choose the optimal number of cell types.

The deconvolved cell-type composition from SpatialCD closely resembled the real spatial patterns from the single cell resolution data (Fig. 2B). To quantify the performance, we compared SpatialCD with STdeconvolve and Spicemix against the ground truth by computing Pearson’s correlation coefficients between the deconvolved cell-type-specific gene expression profiles and the true profiles. SpatialCD consistently outperformed STdeconvolve and Spicemix with higher correlations in both cell-type compositions across all simulated spots (Fig. 2D, Supplemental Fig. S1C), and transcriptional profiles across all genes (Fig. 2F, Supplemental Fig. S1D). We identified the best-matched cell types by checking the similarity through Pearson’s correlation between deconvolved cell types and the ground truth profiles. Generally, SpatialCD showed a higher correlation for nearly all of the cell types (Figs. 2C,E), along with a stronger Spearman's correlation in gene ranking (Fig. 2G, Supplemental Fig. S1E). We observed a strong correlation ( > 0.80) between the cell types deconvolved by SpatialCD and the ground truth composition of astrocytes, ependymal cells, excitatory neurons, and OD immature cells. Particularly, SpatialCD demonstrated a significant improvement in identifying the astrocytes, whereas STdeconvolve struggled to detect this cell type effectively.

Microglia were challenging to identify for both methods due to the restricted MERFISH gene panel, which was originally curated to distinguish neuronal subtypes and includes only limited markers for non-neuronal cell types. Under this constrained feature space, neither method robustly resolved microglia, and the observed differences in correlation metrics between methods were modest and should be interpreted with caution. Still, SpatialCD’s performance of identifying these cells was better than that of STdeconvolve. To further assess the accuracy of cell-type proportion estimates, we computed the root-mean-square error (RMSE) between the deconvolved proportions and the ground truth across all simulated spots. SpatialCD achieved significantly lower RMSE values than both STdeconvolve and Spicemix (Fig. 2H), reflecting more precise and reliable estimates of cell-type composition. This improvement was statistically significant, as confirmed by the Diebold-Mariano test (*p*-value < 2.2 × 10^−16^ ). Although reference-based methods such as RCTD (Cable et al. 2022) can achieve lower RMSE when a well-matched reference is available, this optimal condition is not always guaranteed in practice. When the reference is incomplete or mismatched, for example, missing major neuronal populations or derived from an independent scRNA-seq data, RCTD’s performance degrades substantially, whereas SpatialCD remains robust as it infers transcriptional programs directly from the spatial data (Supplemental Figs. S5, S6). These results highlight the critical role of incorporating spatial information into deconvolution models for improving both the accuracy and spatial coherence of inferred transcriptional patterns.

#### Mouse kidney ST data

**Figure 3.**
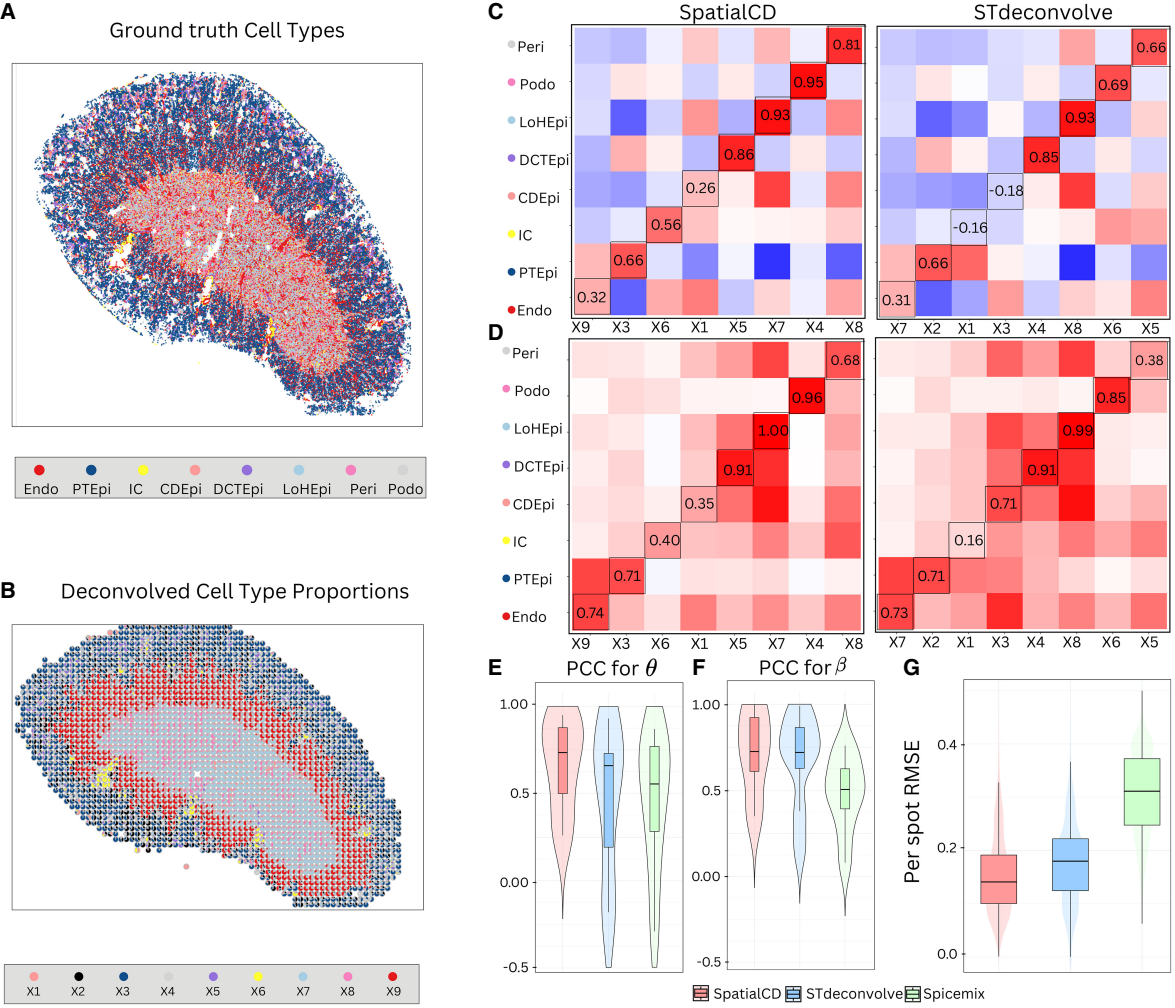
Deconvolution of simulated ST data (MK). (*A*) Ground truth single-cell resolution MERFISH data in mouse kidney. (*B*) Proportions of deconvolved cell types from SpatialCD represented as pie charts for each simulated spot. (*C*) Comparison of cell-type composition (*θ*): Pearson’s correlation heatmaps between deconvolved and ground truth proportions using SpatialCD and STdeconvolve, with matched deconvolved and ground truth cell types highlighted with bounding boxes. (*D*) Comparison of transcriptional profile (*β*): Pearson’s correlation heatmaps between deconvolved and ground truth gene expression profiles using SpatialCD and STdeconvolve, with matched deconvolved and ground truth cell types highlighted. (*E*) PCC for *θ*: Pearson’s correlation coefficients between the deconvolved cell-type composition and matched ground truth cell types across spots for SpatialCD, STdeconvolve, and Spicemix. (*F*) PCC for *β*: Pearson’s correlation coefficients between the deconvolved transcriptional profiles and matched ground truth profiles across genes for SpatialCD, STdeconvolve, and Spicemix. (*G*) Root-mean-square error (RMSE) per spot of the deconvolved cell-type proportions compared to ground truth for SpatialCD, STdeconvolve, and Spicemix. Abbreviations: Endo – endothelial cell; PTEpi – proximal tubule epithelial cell; IC – immune cell; CDEpi – collecting duct epithelial cell; DCTEpi – distal convoluted tubule epithelial cell; LoHEpi – loop of Henle epithelial cell; Peri – pericyte; Podo – podocyte.

We analyzed a publicly available single-cell spatial transcriptomics (ST) data from mouse kidneys (MK) (Liu et al. 2023a), profiled using the Vizgen Multiplexed Error-Robust Fluorescence in situ Hybridization (MERFISH) platform (Moffitt et al. 2018). This MK data set contains the expression of 304 genes from 126,241 cells, annotated into eight distinct cell types. To simulate ST data, we partitioned the single-cell ST data into 2474 spatially contiguous squares, aggregating the gene expression of cells within each square to mimic spots typically observed in ST data (Fig. 3A). The ground truth cell-type proportions, cell-type-specific gene expression profiles, and marker genes were derived from these simulated spots, based on the original single-cell data (Supplemental Fig. S2A).

SpatialCD and STdeconvolve were then applied to this simulated data set to simultaneously infer cell-type composition and transcriptional profiles across the simulated spots. SpatialCD identified *K* = 9 cell types (Supplemental Fig. S2C) whereas STdeconvolve identified *K* = 7 cell types (Supplemental Fig. S2D), and failed to identify one of the cell types listed in the ground truth (Pericyte). To be able to compare with SpatialCD, we fixed the number of cell types as *K* = 8 for both STdeconvolve and Spicemix (Supplemental Fig. S2B).

The deconvolved cell-type composition from SpatialCD closely resembled the real spatial patterns from the single cell resolution data (Fig. 3B). To quantify performance, we compared SpatialCD, STdeconvolve and Spicemix against the ground truth by computing Pearson’s correlation coefficients between the deconvolved cell-type-specific gene expression profiles and the true profiles. SpatialCD consistently yielded more coherent spatial patterns than STdeconvolve and Spicemix, achieving higher correlations for both cell-type compositions across all simulated spots (Fig. 3E, Supplemental Fig. S2E) and transcriptional profiles across all genes (Fig. 3F, Supplemental Fig. S2F). We then compared the performance of SpatialCD and STdeconvolve against the ground truth by computing Pearson’s correlation coefficients between the deconvolved cell-type composition and transcriptional profile to the ground truth (Figs. 3C,D). Notably, whereas STdeconvolve failed to identify immune cell (IC), collecting duct epithelial cell (CDEpi), SpatialCD successfully identified these two cell types and outperformed STdeconvolve with pericyte (Peri), podocyte (Podo) (both Pearson’s correlation coefficients are >0.80). The Spearman's correlations of SpatialCD and STdeconvolve are comparable. As expected, SpatialCD showed significantly lower RMSE per spot for cell-type proportions compared to STdeconvolve and Spicemix, with Diebold-Mariano *p* -values <2.2 × 10^−16^ (Fig. 3G).

In summary, in all simulated ST data sets, SpatialCD consistently yielded more accurate and biologically coherent results than STdeconvolve and Spicemix. This result highlights the importance of incorporating spatial information into deconvolution models.

### SpatialCD characterizes the spatial organization of transcriptionally distinct cell types in real ST data

#### Mouse main olfactory bulb ST data

**Figure 4.**
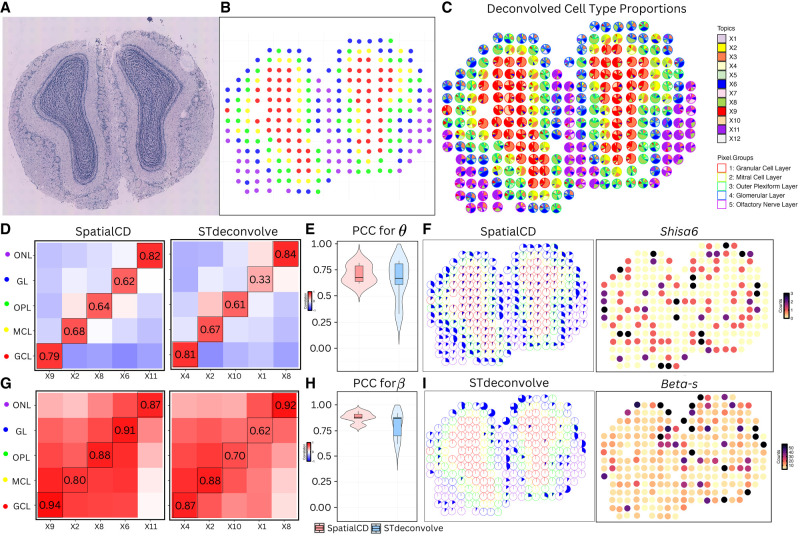
Deconvolution of real ST data (MOB). (*A*) Histology staining image of the mouse main olfactory bulb (MOB) tissue. (*B*) Visualization of spots colored by their transcriptional cluster memberships overlaid on pixel spatial coordinates. (*C*) Proportions of deconvolved cell types from SpatialCD represented as pie charts for each spot. Spots are outlined with colors based on the pixel transcriptional cluster assignment corresponding to MOB coarse cell layers. (*D*) Comparison of cell-type composition (*θ*): Pearson’s correlation heatmaps between deconvolved and ground truth proportions using SpatialCD and STdeconvolve, with matched cell types highlighted with bounding boxes. (*E*) PCC for *θ*: Pearson’s correlation coefficients between deconvolved cell-type compositions and matched ground truth across spots for SpatialCD and STdeconvolve. (*F*) Highlights of the identified deconvolved cell type from SpatialCD (X6) in the glomerular layer (GL) and the corresponding gene counts of its most frequently expressed gene. (*G*) Comparison of transcriptional profile (*β*): Pearson’s correlation heatmaps between deconvolved and ground truth gene expression profiles using SpatialCD and STdeconvolve, with matched cell types highlighted. (*H*) PCC for *β*: Pearson’s correlation coefficients between deconvolved transcriptional profiles and matched ground truth profiles across genes for SpatialCD, STdeconvolve, and Spicemix. (*I*) Highlights of the identified deconvolved cell type from STdeconvolve (X1) in the GL and its most frequent gene. Abbreviations: GCL – Granular Cell Layer; MCL – Mitral Cell Layer; OPL – Outer Plexiform Layer; GL – Glomerular Layer; ONL – Olfactory Nerve Layer.

Having demonstrated that SpatialCD accurately recovers cell-type proportions and transcriptional profiles in simulated ST data, we evaluated its performance on real ST data of the mouse main olfactory bulb (MOB) (Ståhl et al. 2016). This data set contains 12 tissue samples from the same subject, and we focused our analysis on MOB replicate 8 (Fig. 4A), which contains expression count data for 15,928 genes measured across 262 spatial spots. Each spot was annotated into one of five main structural layers based on their transcriptional clustering memberships: granular cell layer (GCL), mitral cell layer (MCL), outer plexiform layer (OPL), glomerular layer (GL) and olfactory nerve layer (ONL) (Fig. 4B).

For fair comparison with STdeconvolve, we used a preprocessed version of the data set consisting of 255 overdispersed genes across 260 spots as the input expression matrix, and set the number of deconvolved cell types to *K* = 12 for all methods (Supplemental Fig. S3A). The cell-type compositions predicted by SpatialCD accurately reflected the layer structure of the MOB (Fig. 4C).

We then computed Pearson’s correlation between the deconvolved cell-type compositions and gene profiles from each method and the known annotated cell layers. SpatialCD consistently yielded more accurate and biologically coherent results than STdeconvolve, achieving higher correlation with the annotated layers and yielding a clearer separation of the five structural regions (Figs. 4E–H; Supplemental Fig. S3B). Notably, while maintaining comparable performance across most layers, SpatialCD showed a marked improvement in accurately resolving the glomerular layer (GL) compared to STdeconvolve. Specifically, the correlation between the ground truth GL and the deconvolved cell-type proportion distributions across all spots from the SpatialCD model is 0.62 (Fig. 4D). Additionally, the correlation between the ground truth GL and the deconvolved cell-type gene expression profiles across all genes is 0.91 (Fig. 4G). Both correlation values are higher than those obtained from the STdeconvolve method, indicating an improved alignment with the ground truth.

Furthermore, when examining the distribution of the selected deconvolved cell type from SpatialCD (X6) (Fig. 4F), this shows a clear and consistent pattern with the ground truth GL layer, and its top expressed gene (*Shisa6*) also demonstrates a strong match with the GL structure. By comparison, the matched deconvolved cell type from STdeconvolve (X1) was only distributed in a few spots (Fig. 4I). The top expressed gene from the high frequency genes within the deconvolved cell type from STdeconvolve (*Beta-s*) also shows a poor alignment with the GL structure (Fig. 4I).

Lastly, the deconvolved cell type results from Spicemix were not comparable, as 9 out of the 12 inferred cell types had zero expression across all spots (Supplemental Fig. S3C), indicating a failure to capture meaningful biological variation in this data set.

These results highlight the critical role of incorporating the spatial graph between neighboring spots in deconvolution models, particularly for detecting thin or transcriptionally subtle layers. By leveraging spatial structure, SpatialCD more effectively captures the layered organization of the MOB and enhances the biological interpretability of the inferred cell-type distributions.

#### Human pancreatic ductal adenocarcinoma

**Figure 5.**
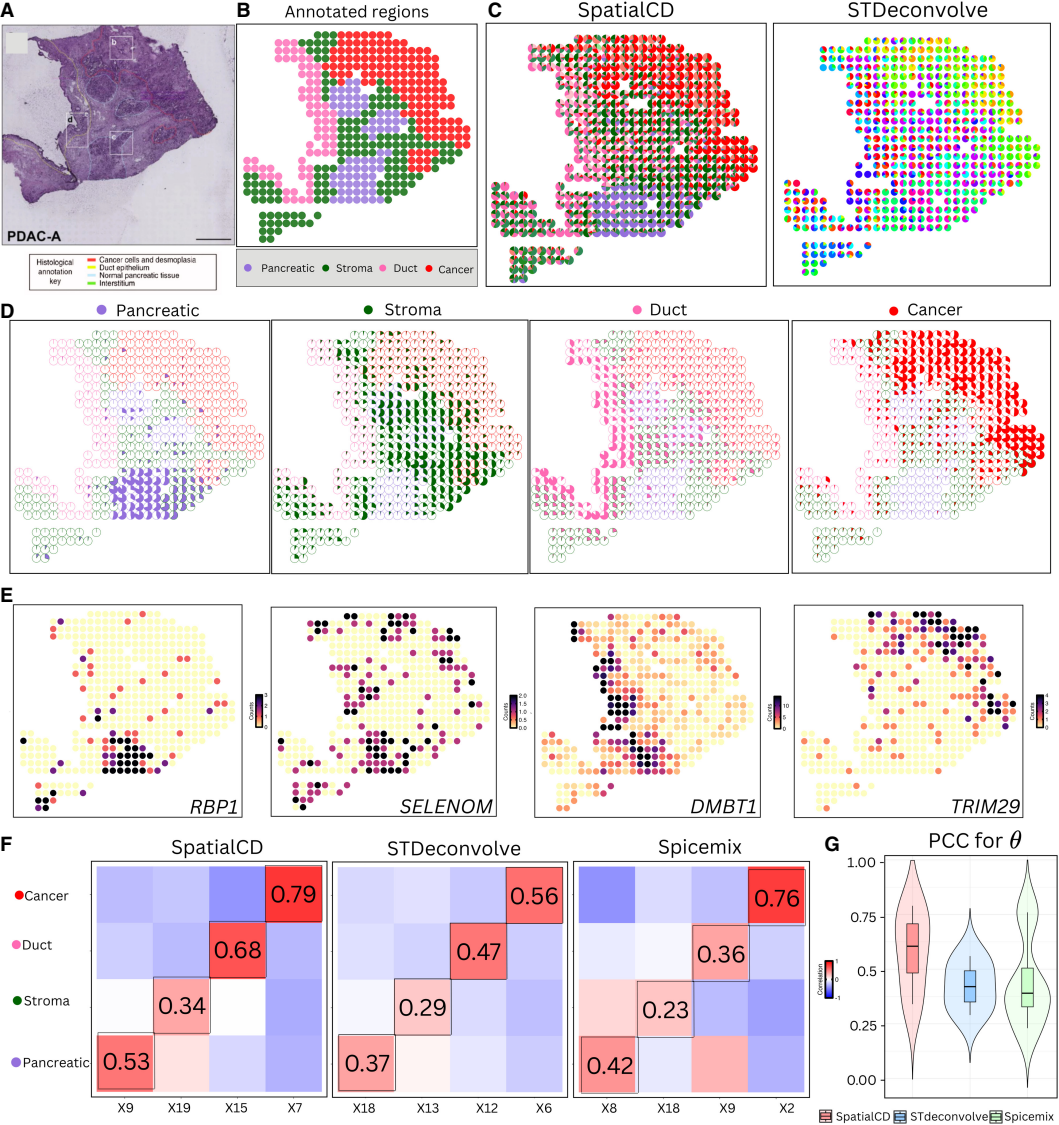
Deconvolution of real ST data (PDAC). (*A*) Histology staining image of human pancreatic ductal adenocarcinoma (PDAC) tissue. (*B*) Visualization of annotated regions defined by histologists from the original study. (*C*) Proportions of deconvolved cell types from SpatialCD and STdeconvolve represented as pie charts for each spot. (*D*) Highlights of identified deconvolved cell types from SpatialCD across annotated regions. (*E*) Gene counts of the top differentially upregulated gene for each deconvolved cell type corresponding to the annotated regions. (*F*) Comparison of cell-type composition (*θ*): Pearson’s correlation heatmaps between deconvolved and ground truth proportions using SpatialCD, STdeconvolve, and Spicemix, with matched deconvolved and ground truth cell types highlighted with bounding boxes. (*G*) PCC for *θ*: Pearson’s correlation coefficients between deconvolved cell-type compositions and matched ground truth across spots for SpatialCD, STdeconvolve, and Spicemix.

In addition to the mouse olfactory bulb tissue ST data set, we further validated the performance of SpatialCD on an ST data set generated from a human pancreatic ductal adenocarcinoma (PDAC) sample. The PDAC data consists of multiple tissue regions (Cancer, Ductal, Pancreatic, and Stroma), which are annotated by a histologist based on the H&E staining image (Figs. 5A,B). We applied the three methods to this data and used 1379 genes (Moncada et al. 2020) from the marker gene list of the PDAC data set as input for deconvolution. As there are 20 cell types listed in the marker gene list, we fixed the number of topics as *K* = 20 for all methods.

As expected, SpatialCD performed the best in identifying the major cell types corresponding to the labeled regions (Fig. 5C). For each best-matched deconvolved cell type, we visualized its spatial distribution by plotting cell-type proportions across spots, with spot boundaries colored according to annotated tissue regions (Fig. 5D). Notably, SpatialCD predicted significantly higher proportions of cancer cells within cancerous regions compared to non-cancerous areas (*t*-test, *p*-value < 2.2 × 10^−16^ , Supplemental Fig. S4A). Similar *t*-tests conducted for other regions yielded consistent results, supporting the ability of SpatialCD to accurately identify region-specific cell types (Supplemental Figs. S4B–D). Additionally, through top gene analysis, SpatialCD accurately identified cell types matching the annotated regions (Fig. 5E). In the pancreatic region, we found *RBP1*, a marker gene for acinar cells. For the stroma region, we found *SELENOM*, which has been reported as a fibroblast-associated marker that is known to be a major cellular component of the stroma in human breast cancer tissue. We identified *DMBT1*, the marker gene for ductal centroacinar cells in the ductal region. Lastly, the marker gene *TRIM29* of Cancer-clone-A was enriched in the deconvolved cell type X7, which is well aligned with the annotated cancer region.

To quantitatively assess the performance, we computed Pearson’s correlation coefficients between the deconvolved cell-type-specific gene expression profiles and the ground truth profiles (Fig. 5G). Notably, SpatialCD achieved higher correlations in identifying cell types associated with all regions, demonstrating the advantage of integrating spatial information for more accurate cell type detection. Compared to STdeconvolve, SpatialCD achieved higher correlations across all regions (Fig. 5F). Although Spicemix competitively identified the cancer region, it performed poorly in other regions (Fig. 5F). Despite these strengths, SpatialCD showed relatively reduced resolution in distinguishing the Stroma and Pancreatic regions compared to its strong performance in identifying tumor-dominated regions. We attribute this to several factors intrinsic to the data rather than model instability. Stromal and pancreatic compartments often share partially overlapping transcriptional programs, particularly in inflamed or tumor-adjacent tissue, making it difficult for latent factor models to form clearly separated components. Furthermore, stromal and pancreatic areas often display gradual spatial transitions and mixed cellular composition rather than sharp boundaries, which can reduce the compositional contrast available to spatially informed models. Users applying SpatialCD to data sets with similar characteristics, such as subtle transcriptional differences or diffuse spatial organization, should interpret results in these regions with appropriate caution.

## Discussion

In this study, we introduced SpatialCD, a reference-free and truly spatially informed deconvolution method for multi-cellular ST data. By extending the LDA framework with spatial regularization, SpatialCD encourages neighboring spots to share similar cell-type compositions, yielding smoother and more biologically coherent deconvolution results without relying on matched scRNA-seq references. This addresses a key limitation of existing deconvolution tools while retaining interpretability and flexibility.

SpatialCD contributes to the growing class of reference-free spatial deconvolution methods. As summarized in recent reviews (Gaspard-Boulinc et al. 2025), reference-based approaches leverage external scRNA-seq data to constrain cell-type inference, whereas reference-free methods rely on factor decomposition of spatial transcriptomics data itself. SpatialCD extends this reference-free paradigm by incorporating explicit spatial graph regularization, thereby addressing the under-constrained nature of factor decomposition through biologically motivated spatial similarity constraint. In practice, applying both reference-based and reference-free deconvolution methods in parallel can provide a useful consistency check, particularly when the quality of the available single-cell reference is uncertain. Reference-based methods such as RCTD perform well when the reference is well matched to the spatial sample, but their accuracy depends critically on this assumption. When reference data are incomplete, mismatched in tissue source or disease state, or lack coverage of rare cell populations, reference-free approaches such as SpatialCD can offer a more robust alternative. Comparing the outputs of the two approaches can help assess reliability: strong concordance suggests that the reference is appropriate, whereas substantial disagreement may indicate reference mismatch and warrant closer inspection of the single-cell atlas.

We evaluated SpatialCD on both simulated and real ST data sets, including simulated mouse brain and kidney data generated from MERFISH, and real tissue samples such as the mouse olfactory bulb (MOB) and pancreatic ductal adenocarcinoma (PDAC). Across diverse simulated and real data sets, SpatialCD consistently yielded more accurate and interpretable results across data sets than STdeconvolve and SpiceMix. The method achieved higher Pearson’s correlations for cell-type compositions and transcriptional profiles, reduced per-spot RMSE, and recovered biologically meaningful spatial patterns, including subtle tissue layers and region-specific tumor-associated cell types. For example, in the MPOA simulation, SpatialCD recovered the correct cell types with higher correlation and successfully identified astrocytes, which STdeconvolve failed to detect. In the MK data set, SpatialCD was able to recover immune cells and collecting duct epithelial cells, whereas STdeconvolve could not. In MOB and PDAC, SpatialCD identified all major cell-type layers and region-specific tumor clones, providing spatially resolved insights not captured by other methods. Compared to STdeconvolve, which models each spot independently, SpatialCD uses spatial information and produces smoother and more coherent results. Although SpiceMix also includes spatial regularization, it is not specifically designed for cell-type deconvolution and does not leverage the strength of the LDA framework for modeling cell-type distribution. Notably, SpatialCD maintains better expression-level accuracy and supports more reliable recovery of gene markers. These results highlight the importance of explicitly incorporating spatial structure into ST deconvolution models.

Although SpatialCD demonstrates notable advantages, several limitations remain. The number of cell types must still be selected through model tuning, and the current framework considers only nearest-neighbor connectivity, which may not fully capture long-range spatial gradients or rare cell populations. Additionally, SpatialCD does not incorporate histology images or multimodal information, and does not yet leverage neural network-based spatial encoders. Future extensions will include integrating spatial histology or H&E image information to improve accuracy and the performance of the deconvolution process.

In addition, gwSPADE (Xie et al. 2025) shows that incorporating gene-level weighting into the LDA framework can improve the discrimination of cell-type-specific transcriptional programs by down-weighting non-informative, high-frequency genes and up-weighting more informative ones. By comparison, SpatialCD focuses on modeling spatial dependence by introducing a graph-based regularization term that encourages similar cell-type compositions across neighboring spots. These two approaches address complementary aspects of reference-free deconvolution: gene-level discrimination versus spatial coherence. Future work may explore combining gene-weighting strategies with spatial regularization to further improve robustness and interpretability.

In summary, SpatialCD offers an interpretable and spatially aware reference-free framework for analyzing ST data. By leveraging spatial graph structure while simultaneously modeling cell-type transcriptional profiles and compositions, SpatialCD advances the methodological toolkit for decoding spatial tissue architecture in the absence of matched single-cell references.

## Methods

### Spatial graph of neighboring spots

To initialize the spatial regularization, SpatialCD starts by constructing an adjacency graph of the *n*_*NN*_ nearest neighbors for each spot. To ensure consistency across different data sets and spatial transcriptomics platforms, we first rescale the spatial coordinates by transforming the original coordinates as: ${\tilde{x}}_{u}=\left(x_{u}-\min_{x}\right)/\left(\max_{x}-\min_{x}\right);{\tilde{y}}_{u}=\left(y_{u}-\min_{y}\right)/\left(\max_{y}-\min_{y}\right)$ where (*x*_*u*_, *y*_*u*_) and $\left({\tilde{x}}_{u},{\tilde{y}}_{u}\right)$ represent the original and normalized coordinates of spot *u*. This transformation helps ensure all coordinates are scaled between 0 and 1. Then, the neighbors of each spot are determined using the Euclidean distance between spots: $d(i,j)=\sqrt{{\left({\tilde{x}}_{i}-{\tilde{x}}_{j}\right)}^{2}+{\left({\tilde{y}}_{i}-{\tilde{y}}_{j}\right)}^{2}}$.

The number of nearest neighbors for each spot, *n*_*NN*_, which is the neighborhood size, is considered as a hyperparameter that influences both spatial graph structure and computational cost. We suggest choosing *n*_*NN*_ based on the spatial arrangement of spots in the ST technology. For example: *n*_*NN*_ = 4 for a “checkerboard” pattern; and *n*_*NN*_ = 6 for a “honeycomb” pattern. By default, SpatialCD sets *n*_*NN*_ = 4, considering the four nearest neighbors for each spot to balance computational efficiency with spatial resolution in the analysis. Users may also adjust the choice of *n*_*NN*_ based on prior knowledge of tissue composition.

### Latent Dirichlet allocation (LDA) model

Latent Dirichlet Allocation (LDA) model (Blei et al. 2003) is a generative probabilistic model originally developed for topic modeling in natural language processing. In the context of spatial transcriptomics, there is a natural analogy: a spatial spot corresponds to a document, genes correspond to words, and latent cell types correspond to topics. Under the LDA framework, each spot is modeled as a mixture of latent cell types, and each cell type is characterized by a probability distribution over genes.

More specifically, LDA assumes that for each spot, cell-type proportions are drawn from a Dirichlet distribution, and gene counts are generated from a multinomial distribution conditioned on these proportions and cell-type-specific gene expression profiles. Importantly, the model does not require predefined reference signatures; instead, both the cell-type-specific gene distributions and spot-level proportions are inferred directly from the observed count data. This enables reference-free deconvolution by leveraging the expression structure of genes across spatial locations.

Formally, let *d* ∈ {1, …, *D*} index spatial spots, and *k* ∈ {1, …, *K*} index latent cell types. Each spot *d* is characterized by a multinomial distribution over *K* latent cell types, denoted by θ_*d*_, and each cell type *k* is characterized by a multinomial distribution over genes *W* , denoted by *β*_*k*_. The generative process for each UMI (unique molecular identifier) *n* in spot *d* is defined as follows:

1. For each spot *d*:
  - Draw cell-type proportions *θ*_*d*_ ∼ Dir(*α*), in which $\theta_{d}\in R^{K}$.
2. For each cell type *k*:
  - Draw gene distribution *β*_*k*_ ∼ Dir(*η*), in which $\beta_{k}\in R^{W}$.
3. For each observed UMI *n* in spot *d*:
  - Draw cell-type assignment *z*_*d*,*n*_ ∼ Multinomial(*θ*_*d*_),
  - Draw gene identity $w_{d,n}∼\text{Multinomial}(\beta_{z_{d,n}})$.

The latent variables in this model include the cell-type proportions $\theta ={\left\{\theta_{d}\right\}}_{d=1}^{D}$, transcriptional profiles $\beta ={\left\{\beta_{k}\right\}}_{k=1}^{K}$, and latent cell-type assignments *z* = {*z*_*d*,*n*_} . Given the observed gene identities *w* = {*w*_*d*,*n*_}, the inference task is to compute the posterior distribution over latent variables, *p*(*z*, *θ*, *β* | *w*, *α*, *η*) . In this study, we utilize variational Bayesian inference (VBI) to approximate this posterior distribution by a simpler distribution *q*(*z*, *θ*, *β*), which is indexed by a set of free parameters (Attias 1999; Jordan et al. 1999). These parameters are optimized to maximize the Evidence Lower BOund (ELBO):

(1) $$\log p(w\;|\;\alpha ,\eta )\geq L(w,\phi ,\gamma ,\lambda )=E_{q}[\log p(w,z,\theta ,\beta \;|\;\alpha ,\eta )]-E_{q}[\log q(w,z,\theta ,\beta )].$$

### ELBO with spatial regularization in SpatialCD

In the standard LDA, all spots are assumed to be independent and unstructured; that is, each spot is assumed to follow a Dirichlet distribution with the same prior hyperparameter *α* = 1/*K*. This is what STdeconvolve assumes in its deconvolution process. In contrast, our framework is designed to incorporate additional information about the nearest neighbors of each spot into the estimation process; that is, neighboring spots are assumed to share similar topic distributions. Using the nearest neighbors graph, in which each spot is a node (spot *i* ) with edges to its nearest neighbors (spot *j*), SpatialCD adds spatial constraints on the Dirichlet prior of the connected spots (*α*_*i*_, *α*_*j*_). Following the idea of the Graph-Fused LASSO regularization (Tansey and Scott 2015), we enforce topic similarity between neighboring spots by penalizing the difference between *α*_*i*_ and *α*_*j*_, and reformulate the ELBO as:

(2) $$L(w,\phi ,\gamma ,\lambda )=E_{q}[\log p(w,z,\theta ,\beta \;|\;\alpha ,\eta )]-E_{q}[\log q(w,z,\theta ,\beta )]-\delta \sum_{(i,j)\in G}w_{ij}{\left\|\alpha_{i}-\alpha_{j}\right\|}_{1},$$

in which *α*_*i*_, *α*_*j*_ are the Dirichlet priors of spots *i* and *j* , G is the spatial graph of neighboring spots, *δ* is a nonnegative tuning parameter, *w*_*ij*_ = 1/*d*_*ij*_ is an edge weight indicating the similarity between connected nodes based on the distance *d*_*ij*_ between nodes.

We denote G=(D,E) an undirected graph induced by the adjacency matrix that captures spatial neighborhood relationships among spots within a sample. The set of nodes D represents the spots, and E denotes the edge set with cardinality *m* = *n*_*NN*_ × *D* , in which *n*_*NN*_ is the number of nearest neighbor for each spot and *D* is the total number of spots in the sample. For simplicity, we assume that G is a binary graph, but our framework can be naturally extended to weighted graphs with each edge weight defined as 1/*d*_*ij*_ , with *d*_*ij*_ denoting the distance between the spot *i* and *j*. Let Λ ∈ *R*^*m*×*D*^ denote the differencing matrix of the graph G. For any edge *E*_*l*_ = (*α*_*i*_, *α*_*j*_) connecting spot *i* and *j* in the graph, the *l* th row of Λ is defined such that Λ_*li*_ = 1, Λ_*lj*_ = −1, and Λ_*ls*_ = 0 for all *s* ≠ *i*, *j* . This construction implies that the rows of the Dirichlet priors matrix of all spot-cell type-priors *α* are assumed to be smooth with respect to the graph G. Let Λ*α* = *e*. Our final objective function ELBO is rewritten as:

(3) $$L(w,\phi ,\gamma ,\lambda )=E_{q}[\log p(w,z,\theta ,\beta \;|\;\alpha ,\eta )]-E_{q}[\log q(w,z,\theta ,\beta )]-\delta \sum_{l=1}^{L}{\left\|e_{l}\right\|}_{1}.$$

### Estimation of cell type composition (*θ*) and gene expression (*β*)

In our framework, the ELBO has been modified as shown in Equation (2), and recall that the main goal here is to maximize this ELBO (*L*) with respect to the variational parameters (*w*, *ϕ*, *γ*, *λ*) and *α*. To do so, we follow onlineLDA, an online variational Bayes (VB) algorithm for LDA developed by Hoffman et al. (2010). By analogy to the Expectation-Maximization (EM) algorithm (Dempster et al. 1977), we can partition these updates into an “E” step: iteratively updating *γ* and *ϕ* until convergence, while holding other parameters fixed; and an “M” step: updating *λ* given *ϕ*. Finally, we can iterate to update α given all variational parameters of LDA. This optimization, however, is challenging due to the presence of a non-differentiable *l*_1_ penalty with a nonlinear Dirichlet parameterization. To address this, we adopt a strategy inspired by the graph-fused LASSO formulation in Tansey and Scott (2015), and reformulate the problem using a Lagrangian dual decomposition. We solve this problem efficiently using the Alternating Direction Method of Multipliers (ADMM) (Boyd et al. 2011). Our algorithm is presented in Algorithm 1, and the detailed derivations are provided in the Supplemental Materials. As a result, the model yields estimates of the cell-type proportion matrix *θ*_*d*_ for each spot *d* represented by *γ*_*d*_, and the gene expression matrix *β*_*k*_ for each cell type *k* represented by *λ*_*k*_, which are then used for downstream analyses.

Algorithm 1. **Online Variational Inference for SpatialCD**

 Initialize λ randomly

Updating VB parameters of LDA:

**for** *t* = 0 to ∞ **do**

  E-step:

  Initialize $\gamma_{t}\leftarrow \;1$

  **repeat**

   **for** each gene *w* = 1 to *W* **do**

    **for** each topic *k* = 1 to *K* **do**

     $\phi_{twk}\propto \exp(E_{q}[\log\theta_{tk}]+E_{q}[\log\beta_{kw}])$ (Hoffman et al. 2010)

    **end for**

   **end for**

   **for** each topic *k* = 1 to *K* **do**

    $\gamma_{tk}\leftarrow \alpha_{tk}+\sum_{w=1}^{W}n_{tw}\phi_{twk}$ (Hoffman et al. 2010)

   **end for**

  **until** convergence of γ_*t*_

  M-step:

  **for** each topic *k* = 1 to *K* **do**

   **for** each gene *w* = 1 to *W* **do**

    ${\tilde{\lambda }}_{kw}\leftarrow \eta +D\cdot n_{tw}\cdot \phi_{twk}$ (Hoffman et al. 2010)

    $\lambda_{kw}^{\left(t+1\right)}\leftarrow (1-\rho_{t})\lambda_{kw}^{\left(t\right)}+\rho_{t}{\tilde{\lambda }}_{kw}$

   **end for**

  **end for**

 **end for**

 Update α_*dk*_:

 **for** each spot *d* = 1 to *D* **do**

  **for** each topic *k* = 1 to *K* **do**

   Update **α**^(*k*)^, **e**^(*k*)^, **u**^(*k*+1)^ via primal-dual interior-point method

   Update **τ**^(*k*+1)^ via Newton-Raphson method

   Update dual variables **v**^(*k*+1)^, **z**^(*k*)^, and **r**^(*k*)^

  **end for**

 **end for**

### Deconvolution analysis evaluation

#### Data preprocessing

To apply SpatialCD, ST data, including a gene expression matrix and associated spatial coordinates, is required. The spatial gene expression data is a *D* × *N* matrix of unique molecular identifier (UMI) counts with *D* spots and *N* genes, along with the (*x*, *y* ) two-dimensional (2D) spatial coordinates of each spot. Before running the deconvolution model, the following data preprocessing steps are implemented to improve robustness and interpretability. First, genes with insufficient variability are filtered out: those expressed in fewer than 5% or in all (100%) of the spatial spots are removed. This step reduces noise and improves the model’s ability to detect meaningful patterns. Then we selected the significantly overdispersed genes, or genes exhibiting greater variability in expression across spatial locations than would be expected by chance. These genes are likely to reflect spatially varying cell-type proportions, making them more informative for deconvolution. As ST data can contain thousands of genes, using too many can hinder the model’s ability to learn well-separated transcriptional patterns. To mitigate this, we limit the analysis to the top 1000 overdispersed genes by default, as this threshold generally provides stable deconvolution performance. Additional gene filtering or cell-type-specific marker genes to include in the input ST data may also be augmented by the user as needed (Miller et al. 2022).

#### Selection of LDA model with optimal number of cell-types

Following the approach used in STdeconvolve, we perform a grid search over candidate values of *K* , the number of cell types, to identify the most appropriate model for a given spatial transcriptomics data set. For each candidate *K* , we fit an LDA model and evaluate its performance using perplexity, a standard metric from information theory that quantifies how well a probabilistic model explains the observed data. Lower perplexity values indicate better model fit. The perplexity for a data set *D* is computed as:

$$\mathrm{Perplexity}(D)=\exp\left\{-\frac{\log(p(D))}{\sum_{d=1}^{D}\sum_{n=1}^{N}c_{d,n}}\right\},$$

in which *p*(*D*) is the posterior likelihood of the data set conditional on the cell-type assignments using the final estimated *θ* and *β* and *c*_*d*,*n*_ is the gene expression level, of gene *n* in pixel *d* . In addition to perplexity, we monitor the number of deconvolved cell types whose average proportion across all spatial spots is <5% (as default). As *K* increases, the number of such rare components tends to rise, potentially reflecting overfitting or artificial splits of major cell types. To strike a balance between model complexity and interpretability, we select the largest *K* value that achieves low perplexity while minimizing the number of rare cell types. This joint criterion helps avoid underfitting (too few cell types) and overfitting (too many indistinct or biologically irrelevant components). Users may also adjust the choice of *K* based on prior biological knowledge of tissue composition (Miller et al. 2022).

#### Annotation and matching of deconvolved and ground truth cell types

For the simulated ST data sets, to evaluate how well each deconvolved cell type aligned with the ground truth, we first computed Pearson’s correlation between their transcriptional profiles. For every deconvolved cell type, we identified the ground truth cell type with the highest correlation, based on a full pairwise comparison between all deconvolved and ground truth profiles. For the real ST data sets, we validated the deconvolved cell-type assignments by checking whether the deconvolved transcriptional profiles were enriched for genes that are specifically expressed in the corresponding ground truth cell type. To define these marker genes, the ground truth transcriptional profiles were normalized to counts per thousand. Genes with low expression (mean expression < 5) were excluded. For each ground truth cell type, we calculated the log_2_ fold change of each gene relative to the average expression across all other cell types. Genes with a log_2_ fold change greater than 1 were considered differentially expressed, highlighting the unique transcriptional profile of each deconvolved cell type. Next, we used rank-based gene set enrichment analysis (GSEA), implemented in the R package liger (Fan 2019), to test whether these top-expressed genes were enriched in the transcriptional profile of each deconvolved cell type.

#### Comparison of deconvolution models

To compare the performance between deconvolution methods, we evaluated Pearson’s correlation for both deconvolved cell-type expression profiles and cell-type composition for each pair of ground truth cell types and the matched deconvolved cell type across all spots. Higher correlation indicates better matching to the ground truth cell types, and more similar composition distribution across spots. We further calculated the root mean square error (RMSE) to assess the accuracy of the deconvolved cell-type composition within each spot. For each spot, RMSE between the deconvolved and matched ground truth cell-type proportions is calculated as:

$$\mathrm{RMSE}=\sqrt{\frac{\sum_{k=1}^{K}{\left(\hat{\theta_{k}}-\theta_{k}\right)}^{2}}{K}},$$

in which *K* is the number of cell types,$\hat{\theta_{k}}$ is the deconvolved cell-type proportion for cell type *k*, and *θ*_*k*_ is the ground truth cell-type proportion for cell type *k*. To assess whether the distribution of per-spot RMSEs was significantly lower for SpatialCD compared to other methods, we used a one-sided Diebold–Mariano test.

### Code availability

The Python code to implement SpatialCD is available at GitHub (https://github.com/Cui-STT-Lab/SpatialCD) and as Supplemental Code.

## Competing interest statement

The authors declare no competing interests.

## Supplemental Material

Supplement 1

Supplement 2
