# Supplementary figures and images for: Spatially informed reference-free cell-type deconvolution for spatial transcriptomics with SpatialCD
Source: Genome Res. 2026 Jul;36(7):1455–65. doi: 10.1101/gr.281829.125 (PMC13322189; doi:10.1101/gr.281829.125)

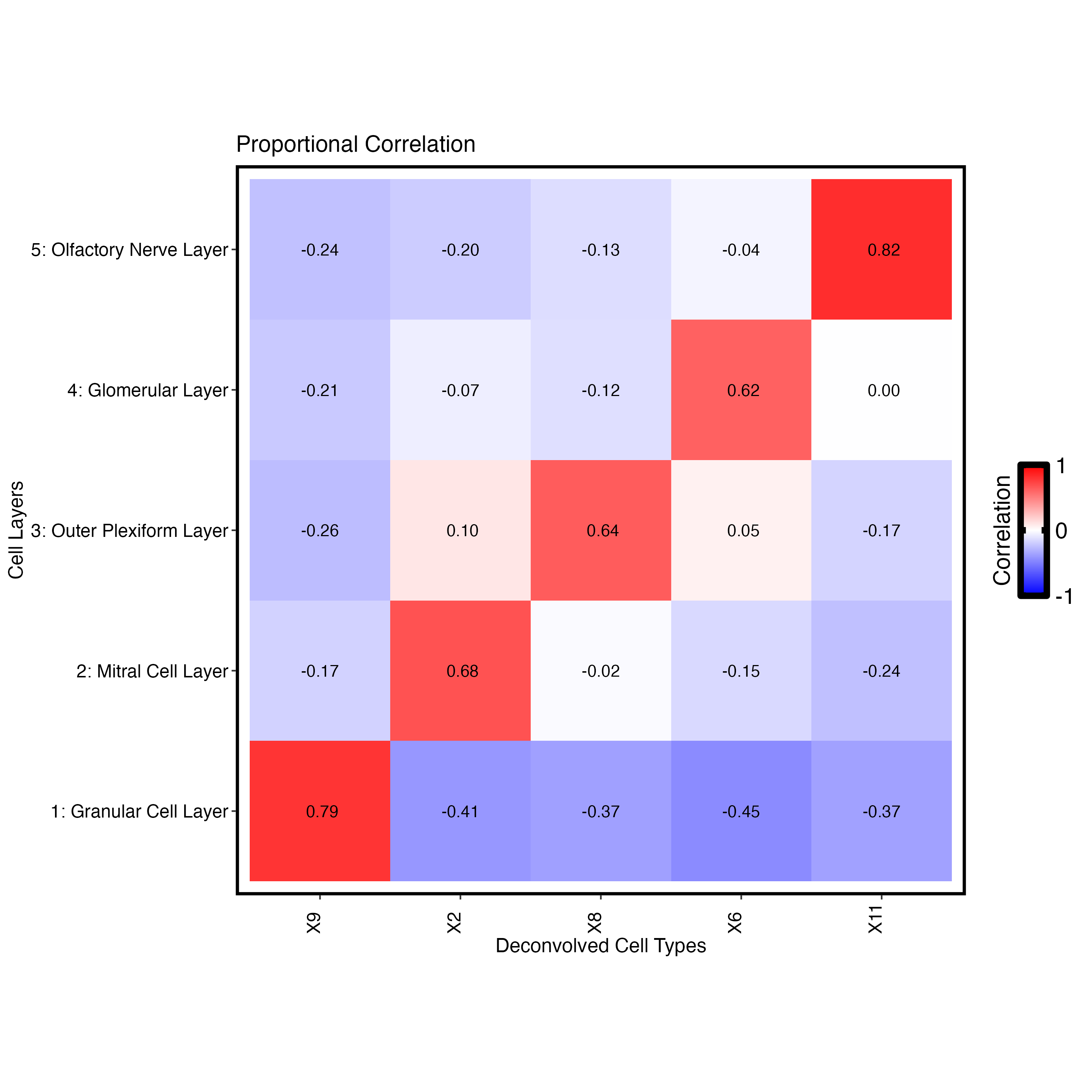

Supplement: Supplement 1 [file Supplemental_Code.zip › SpatialCD-main/example/output/mob/heatmap_mob.png]

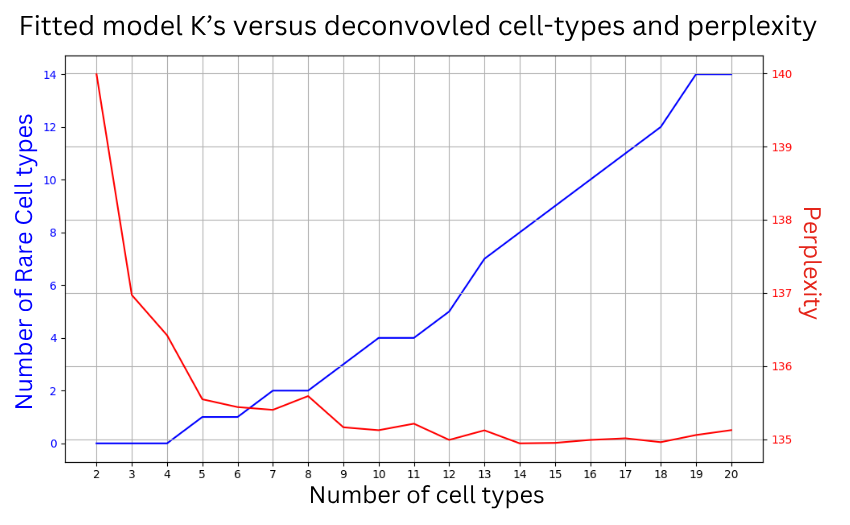

Supplement: Supplement 1 [file Supplemental_Code.zip › SpatialCD-main/example/output/mob/ppxt_mob.png]

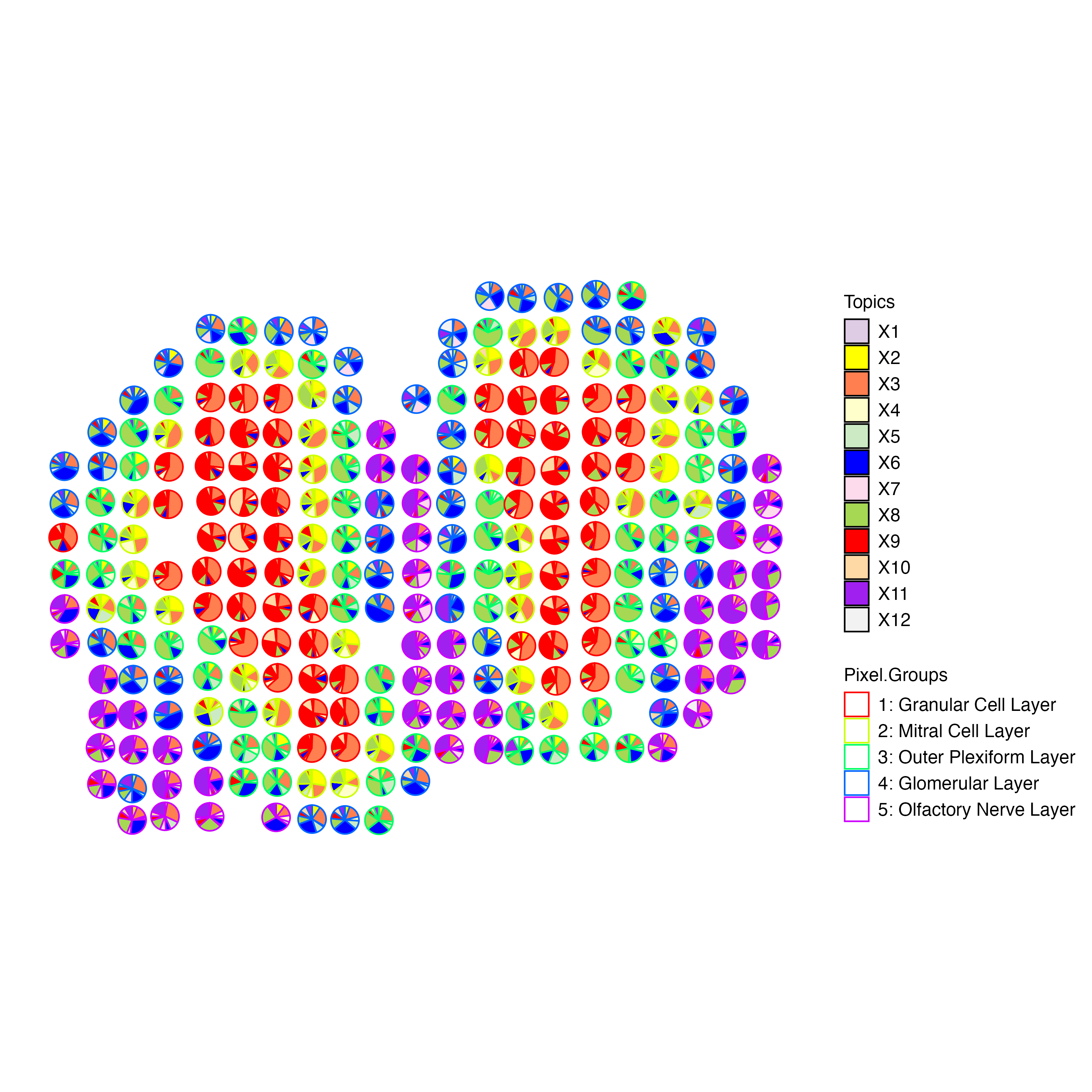

Supplement: Supplement 1 [file Supplemental_Code.zip › SpatialCD-main/example/output/mob/spatialplt_mob.png]
