# Supplementary material for: Spatially informed reference-free cell-type deconvolution for spatial transcriptomics with SpatialCD
Source: Genome Res. 2026 Jul;36(7):1455–65. doi: 10.1101/gr.281829.125 (PMC13322189; doi:10.1101/gr.281829.125)
Supplement: Supplement 2 [file Supplemental_Material.pdf]

Supplementary Material for Spatially informed reference-free cell type deconvolution for spatial transcriptomics with SpatialCD

Phuong Vo[1] and Yuehua Cui*[1]

[1]Department of Statistics and Probability, Michigan State University, East Lansing, MI, 48824, USA

April 30, 2026

This supplementary file contains supplementary figures, methods, and additional analyses supporting the main manuscript.

1 Additional Methodological Details of SpatialCD

1.1 Probabilistic Framework and Biological Analogy of Latent Dirichlet Allocation (LDA) Model

SpatialCD is based on the Latent Dirichlet Allocation (LDA) framework (Blei et al. 2003), which provides a probabilistic foundation for reference-free deconvolution of spatial transcriptomics (ST) data. In this framework, each spatial spot is modeled as a mixture of latent cell types, and each cell type is represented by a distribution over genes. Both the cell-type proportions (θ) and the cell-type-specific gene expression profiles (β) are inferred directly from the observed gene count data, without requiring predefined reference signatures. This is enabled by the ability of LDA to capture recurring gene expression patterns across spatial locations: genes that consistently co-occur across subsets of spots are grouped into latent components, which correspond to putative cell types.

In the context of ST data, topic modeling terminology admits a natural biological analogy. Each spatial spot can be viewed as a "document" composed of a mixture of latent cell types (analogous to "topics"), while genes correspond to "words", and gene counts reflect their observed frequencies within each spot. Each latent topic corresponds to a cell type, characterized by a probability distribution over genes that reflects its transcriptional profile. The topic proportions in a document correspond to the relative abundance of cell types within a spot. Under this analogy, the observed gene expression in each spot arises from a mixture of cell-type-specific transcriptional programs, analogous to how a document is generated from a mixture of topics with distinct vocabularies.

The interpretability of the inferred cell types arises from the structure of the model. Each latent component is associated with a gene distribution that emphasizes genes with higher probability within that component, thereby capturing characteristic transcriptional programs. Meanwhile, variation in cell-type composition across spatial locations provides the heterogeneity necessary to distinguish these components. From a biological perspective, this reflects the assumption that distinct cell types exhibit consistent gene

*Corresponding author: cuiy@msu.edu

expression patterns and are unevenly distributed across tissue. As a result, LDA enables the recovery of biologically meaningful cell-type-specific gene expression profiles together with their spatial proportions in a fully unsupervised manner.

1.2 Variational Bayes Inference (VBI) in SpatialCD

As mentioned in Section 3.4 of the main text, the main objective of SpatialCD is to maximize the ELBO below with respect to the variational parameters $(w, \phi, \gamma, \lambda)$ and the prior parameter α:

$$L(w, \phi, \gamma, \lambda) = \mathbb{E}_q[\log p(w, z, \theta, \beta|\alpha, \eta)] - \mathbb{E}_q[\log q(w, z, \theta, \beta)] - \delta \sum_{l=1}^{L} \|e_l\|_1 \tag{1}$$

To do so, we will use *coordinate ascent*, in which we maximize L with respect to one set of parameters, keeping the others fixed. We then alternate to another set of variables, keeping others fixed, and so on. First, we further compute each terms and (1) is written as a function of variational parameters:

$$\begin{aligned} L(.) = \sum_{d=1}^{D} \Bigg\{ & \sum_{w=1}^{W} n_{dw} \sum_{k=1}^{K} \phi_{dwk} (\mathbb{E}_q[\log \theta_{dk}] + \mathbb{E}_q[\log \beta_{kw}] - \log \phi_{dwk}) \\ & - \log \Gamma \left(\sum_{k=1}^{K} \gamma_{dk} \right) + \sum_{k=1}^{K} \left(\log \Gamma(\gamma_{dk}) + (\alpha_{dk} - \gamma_{dk}) \mathbb{E}_q[\log \theta_{dk}] \right) \Bigg\} \\ & + \sum_{k=1}^{K} \left\{ - \log \Gamma \left(\sum_{k=1}^{K} \lambda_{kw} \right) + \sum_{w=1}^{W} \left(\log \Gamma(\lambda_{kw}) + (\eta - \lambda_{kw}) \mathbb{E}_q[\log \beta_{kw}] \right) \right\} \\ & + D \left[\log \Gamma \left(\sum_{k=1}^{K} \alpha_{dk} \right) - \sum_{k=1}^{K} (\log \Gamma(\gamma_{dk}) \right] + K[\log \Gamma(W\eta) - W \log \Gamma(\eta)] - \delta \sum_{l=1}^{L} \|e_l\|_1 \end{aligned} \tag{2}$$

Maximizing with respect to ϕ

By only keeping the terms involving ϕ, and treating everything else as constants, we then solve the optimizing problem of the objective function L in (2) w.r.t. ϕ to update ϕ:

$$\phi_{dwk} \propto exp\{\mathbb{E}_q[\log \theta_{dk}] + \mathbb{E}_q[\log \beta_{kw}]\} \tag{3}$$

We can further write them in more details by computing the expectations:

$$\mathbb{E}_q[\log \theta_{dk}] = \Psi(\gamma_{dk}) - \Psi \left(\sum_{i=1}^{K} \gamma_{di} \right)$$

$$\mathbb{E}_q[\log \beta_{kw}] = \Psi(\lambda_{kw}) - \Psi \left(\sum_{j=1}^{W} \lambda_{wj} \right)$$

Maximizing with respect to γ

Similarly, we maximize L in (2) with respect to γ:

$$\gamma_{dk} = \alpha_{dk} + \sum_{w=1}^{W} n_{dw} \phi_{dwk} \tag{4}$$

Maximizing with respect to λ

Similarly, we maximize L in (2) with respect to λ:

$$\lambda_{kw} = \eta + \sum_{d=1}^{D} n_{dw}\phi_{dwk} \tag{5}$$

Maximizing with respect to α

To update α, we separate the terms in the ELBO in (2) that involve α, treating all other terms as constants. Then, we minimize the following objective function:

$$L(\cdot) = \frac{1}{D}\sum_{i=1}^{D} \log \frac{\prod_k \Gamma(\alpha_{ik})}{\Gamma(\sum_{k=1}^{K}\alpha_{ik})} - \sum_{i=1}^{D}\sum_{k=1}^{K}(\alpha_{ik}-1)\mathbb{E}_q[\log\theta_{dk}] + \delta\sum_{l=1}^{L}\|e_l\|_1 \tag{6}$$

This optimization, however, is challenging due to the presence of a non-differentiable l_1 penalty with a nonlinear Dirichlet parameterization. To address this, we adopt a strategy inspired by the graph-fused lasso formulation in (Tansey and Scott 2015), and reformulate the problem using a Lagrangian dual decomposition. Specifically, we rewrite the constrained optimization as:

$$\min_{\alpha,\, e}\left\{\frac{1}{D}\sum_{i=1}^{D}\log\left(\frac{\prod_{k=1}^{K}\Gamma(\alpha_{ik})}{\Gamma\left(\sum_{k=1}^{K}\alpha_{ik}\right)}\right) - \sum_{i=1}^{D}\sum_{k=1}^{K}\alpha_{ik}\,\mathbb{E}_q[\log\theta_{dk}] + \delta\sum_{l=1}^{L}\|e_l\|_1\right\}$$
$$\text{subject to} \quad e_l = \alpha_i - \alpha_j, \quad \text{for } (i,j)\in\mathcal{G},\ l = 1,\ldots,L,$$

We solve this problem efficiently using the Alternating Direction Method of Multipliers (ADMM) (Boyd et al. 2011). This approach introduces auxiliary variables to separate the smooth and non-smooth components of the objective, converting the l_1 penalty into linear constraints. By alternating updates over the primal and dual variables, ADMM enables scalable optimization while maintaining convergence guarantees.

$$\min_{\alpha,\tau,e}\left\{\frac{1}{D}\sum_{i=1}^{D}\log B(\tau_i) - \frac{1}{D}\sum_{i=1}^{D}\tau_i^T c_i + \delta\sum_{l=1}^{L}\|e_l\|_1\right\}$$
$$\text{subject to } \tau = \alpha$$
$$\Lambda\alpha = e$$

where $B(\tau_i) = \frac{\prod_{k=1}^{K}\Gamma(\tau_{ik})}{\Gamma(\sum_{k=1}^{K}\tau_{ik})}$; $c_i = \mathbb{E}_q[\log\theta_{dk}]$; Λ is the differencing matrix defined in the main text. Apply Augmented Lagrangian Equation (Boyd et al. 2011), the objective function is rewritten as:

$$\mathcal{L}(\boldsymbol{\alpha}, e, \mathbf{u}, \mathbf{v}) = \left\{\frac{1}{D}\sum_{i=1}^{D}\left[\log B(\tau_i) - \boldsymbol{\tau}_i^T\mathbf{c}_i\right] + \sum_{l=1}^{L}\boldsymbol{\delta}^T e_l - \mathbf{u}_1^T(e-\Lambda\alpha) - \mathbf{u}_2^T(e+\Lambda\alpha)\right.$$
$$\left. - \mathbf{v}^T(\boldsymbol{\tau}-\boldsymbol{\alpha}) + \frac{\rho}{2}\|\boldsymbol{\tau}-\boldsymbol{\alpha}\|_2^2\right\} \tag{7}$$

Using the method of multipliers (Boyd et al. 2011), we can solve this problem with the following updates:

$$\alpha^{(k)},\, e^{(k)},\, \mathbf{u}^{(k+1)} = \arg\min_{\alpha,\, e}\, \max_{\mathbf{u}} \left\{ \mathcal{L}(\alpha, e, \mathbf{u}) + \frac{\rho}{2} \left\| \alpha - \mathbf{z}^{(k)} \right\|_2^2 \right\} \tag{8}$$

$$\boldsymbol{\tau}^{(k+1)} = \arg\min_{\boldsymbol{\tau}} \left\{ \mathcal{L}(\boldsymbol{\tau}) + \frac{\rho}{2} \left\| \boldsymbol{\tau} - \mathbf{r}^{(k)} \right\|_2^2 \right\} \tag{9}$$

$$\mathbf{z}^{(k)} = \boldsymbol{\tau}^{(k)} + \frac{1}{\rho}\mathbf{v}^{(k)} \tag{10}$$

$$\mathbf{r}^{(k)} = \alpha^{(k+1)} - \frac{1}{\rho}\mathbf{v}^{(k)} + \frac{1}{\rho}\mathbf{c} \tag{11}$$

$$\mathbf{v}^{(k+1)} = \mathbf{v}^{(k)} + \rho \left(\boldsymbol{\tau}^{(k+1)} - \alpha^{(k+1)} \right) \tag{12}$$

where $\mathbf{v}$ is the scaled dual variable, ρ is the scalar penalty parameter, and $\mathbf{u} = (\mathbf{u}_1^T, \mathbf{u}_2^T)$ is the Lagrange multipliers. To solve for the updates defined in Equations (8), we apply the primal-dual interior-point methods (Boyd and Vandenberghe 2004) using the modified Karush-Kuhn-Tucker (KKT) conditions expressed as $r_t(\alpha, e, u) = 0$, where we define:

$$r_t(\alpha, e, u) = \begin{bmatrix} \nabla f_0(\boldsymbol{\alpha}, e) + Df(\boldsymbol{\alpha}, e)^T \mathbf{u} \\ -diag(\mathbf{u}) f(\boldsymbol{\alpha}, e) - 1/t\mathbf{1} \end{bmatrix}$$

and $t > 0$ is the central path parameter. The function $f : \mathbf{R}^n \to \mathbf{R}^m$ represents the inequality constraints, and its Jacobian matrix Df is given by

$$f(.) = \begin{bmatrix} f_1(.) \\ . \\ . \\ . \\ f_m(.) \end{bmatrix}, Df(.) = \begin{bmatrix} \nabla f_1(.)^T \\ . \\ . \\ . \\ \nabla f_m(.)^T \end{bmatrix}.$$

The residual vector r_t consists of two blocks:

- The dual residual: $r_{dual} = \nabla f_0(\boldsymbol{\alpha}, e) + Df(\boldsymbol{\alpha}, e)^T \mathbf{u}$,
- The centrality residual: $r_{cent} = -diag(\mathbf{u}) f(\boldsymbol{\alpha}, e) - 1/t\mathbf{1}$.

The Newton step is then computed by solving the following linear system:

$$\begin{bmatrix} \nabla f_0(\boldsymbol{\alpha}, e) + Df(\boldsymbol{\alpha}, e)^T \mathbf{u} \\ -diag(\mathbf{u}) f(\boldsymbol{\alpha}, e) - 1/t\mathbf{1} \end{bmatrix} \begin{bmatrix} \Delta(\boldsymbol{\alpha}, e) \\ \Delta\mathbf{u} \end{bmatrix} = - \begin{bmatrix} r_{dual} \\ r_{cent} \end{bmatrix} \tag{13}$$

While the full details of constructing and solving this system are omitted, we employ a sparse linear solver to compute the Newton step directions. A backtracking line search is then performed to determine an appropriate step size. To solve for the update defined in Equations (9), we apply the linear-time Newton-Raphson method (**?**):

$$\boldsymbol{\tau}_{new} = \boldsymbol{\tau}_{old} - H(\boldsymbol{\tau}_{old})^{-1} g(\boldsymbol{\tau}_{old}) \tag{14}$$

where $g(\boldsymbol{\tau})$ and $H(\boldsymbol{\tau})$ are the gradient and Hessian matrix of the objective function at the point $\boldsymbol{\tau}$:

$$\mathbf{g}(\boldsymbol{\tau}) = \Psi(\boldsymbol{\tau}) - \mathbf{1} \cdot \Psi \left(\sum_{k=1}^{K} \tau_k \right) + \rho \left(\boldsymbol{\tau} - \mathbf{r} \right),$$

$$\mathbf{H} = \text{diag} \left(\Psi'(\boldsymbol{\tau}) + \rho \right) + \mathbf{1}\mathbf{1}^T \Psi' \left(\sum_{k=1}^{K} \tau_k \right),$$

where $\Psi(.)$ and $\Psi'(.)$ denote the digamma and trigamma functions, respectively. To avoid explicit matrix inversion, we use the Sherman–Morrison–Woodbury identity to compute $\mathbf{H}^{-1}\mathbf{g}$ in closed form:

$$(\mathbf{H}^{-1}\mathbf{g})k = \frac{g_k - b}{qkk}, \text{where } b = \frac{\sum_j g_j/q_{jj}}{1/z + \sum_j 1/q_{jj}},$$

1.3 Bayesian Interpretation of Spatial Regularization

Adding regularization terms to an objective function is common in machine learning. For a parameter vector $\mathbf{w}$, a generic regularized loss can be written as

$$L(\mathbf{w}) = l(\mathbf{w}) + r(\mathbf{w}),$$

where $l(\mathbf{w})$ is a data-fitting term and $r(\mathbf{w})$ is a penalty (Wolinski et al. 2020). From a Bayesian perspective, this corresponds to Maximum A Posteriori (MAP) estimation with a prior proportional to $\exp(-r)$ (MacKay 1992). That is,

$$L_{\text{MAP}}(\mathbf{w}) = l(\mathbf{w}) - \ln \alpha(\mathbf{w}), \quad \text{with } \alpha(\mathbf{w}) \propto \exp(-r(\mathbf{w})).$$

In practice, standard LDA is most often estimated via Variational Inference (VI) rather than MAP. VI seeks a variational distribution $q_{\mathbf{u}}(\mathbf{w})$ from a tractable family, parameterized by $\mathbf{u}$, that approximates the true posterior $p(\mathbf{w} \mid \text{data})$. The penalty–prior equivalence also carries over to VI, with priors again corresponding to exponential forms of penalty terms (Wolinski et al. 2020).

To incorporate spatial information, SpatialCD introduces a generalized fused Lasso (Tansey and Scott 2015) like penalty on the Dirichlet priors α_d of spot-level topic proportions to encourage smoothness. Specifically,

$$r(\cdot) = \delta \sum_{(i,j)\in\mathcal{G}} w_{ij}\|\alpha_i - \alpha_j\|_1 = \delta \sum_{l=1}^{L} \|e_l\|_1,$$

where $e_l = \alpha_i - \alpha_j$ for edge $(i, j) \in \mathcal{G}$, L is the total number of edges, and $\delta > 0$ is a tuning hyperparameter. In a Bayesian formulation, this penalty corresponds to Laplace (double-exponential) priors on each e_l, i.e.,

$$\alpha(e|\delta) \propto \exp(-\delta \sum_{l=1}^{L} \|e_l\|_1).$$

The additivity property of Kullback–Leibler divergences ensures that such penalties decompose into independent priors over the edge differences.

The original variational objective function for LDA is the Evidence Lower Bound (ELBO), i.e.,

$$L(w, \phi, \gamma, \lambda) = \mathbb{E}_q[\log p(w, z, \theta, \beta \mid \alpha, \eta)] - \mathbb{E}_q[\log q(w, z, \theta, \beta)]$$

where $q(\cdot)$ is a chosen variational posterior. With spatial regularization, the ELBO becomes

$$L(w, \phi, \gamma, \lambda, e) = \mathbb{E}_q[\log p(w, z, \theta, \beta \mid \alpha, \eta)] - \mathbb{E}_q[\log q(w, z, \theta, \beta)] - \delta \sum_{l=1}^{L} \|e_l\|_1. \tag{15}$$

Empirically, δ is chosen in the range 0.01–0.1. This spatially aware formulation enforces smoothness of topic proportions across neighboring spots while preserving the ability to capture sharp boundaries in cell-type composition.

2 Simulating ST data

2.1 Mouse Medial Preoptic Area (MPOA)

MERFISH data of the mouse medial preoptic area (MPOA) was obtained from the original publication (Moffitt et al. 2018) and is available to download from `https://datadryad.org/stash/dataset/doi:10.5061/dryad.8t8s248`. Following the simulation procedure provied by STdeconvolve (Miller et al. 2022), normalized gene expression values were converted back to counts by dividing by 1000 and multiplying by each cell's absolute volume. Datasets for an untreated female animal containing counts for 135 genes assayed by MERFISH were used. Genes with non-count expression intensities assayed by sequential FISH were omitted. Counts of blank control measurements were also removed. Cells were previously annotated as being one of 9 major cell types (astrocyte, endothelial, microglia, immature or mature oligodendrocyte, ependy- mal, pericyte, inhibitory neuron, excitatory neuron). Cells originally annotated as "ambiguous" were removed from the dataset to ensure the ground truth was composed of cells with distinguishable cell types. Because certain cell types may be enriched in specific regions of the MPOA, we combined 12 tissue sections across the anterior and posterior regions to ensure that all expected cell types would be well represented in the final simulated ST dataset. After filtering, the final dataset contained 59,651 cells representing 9 total cell types and counts for the 135 genes. To simulate a multi-cellular pixel resolution ST dataset from such single-cell resolution spatially resolved MERFISH data, we generated a grid of squares, each square with an area of 100 μm^2. Each square was considered a simulated pixel and the gene counts of cells whose x–y centroid was located within the coordinates of a square pixel were summed together. A grid of square pixels was generated for each of the 12 tissue sections separately and the simulated pixels for all 12 tissue sections were subsequently combined into a single ST dataset. For a given tissue section, the bottom edge of the grid was the lowest y-coordinate of the cell centroids and the left edge of the grid was the lowest x-coordinate. Square boundaries were then drawn from each of these edges in 100 μm^2 increments until the position of the farthest increment from the origin was greater than the highest respective cell centroid coordinate. After generating the grid, square pixels whose edges formed one of the outside edges of the grid were discarded in order to remove simulated pixels, which by virtue of their placement, encompassed space outside of the actual tissue sample. The retained pixels covered 49,142 out of the original 59,651 cells in the 12 tissue sections. This resulted in a simulated ST dataset with 3,072 pixels by 135 genes. We used the original cell type labels of each cell to compute the ground truth proportions in each simulated pixel. Likewise, to generate the ground truth transcriptional profiles of each cell type, we averaged the gene counts for cells of the same cell type from the original 59,651 cells and normalized the resulting gene count matrix to sum to 1 for each cell type. We fitted the SpatialCD and STdeconvolve models using the same count matrix with number of topics K as integer values from 2 to 20 and selected $K = 12$, which minimized perplexity and resulted in fewer rare cell types with mean spot proportions below 5%, which are $K = 12$ for both. Since SpiceMix model is not able to find the optimized number of topics K, we fixed $K = 9$, the same as the number of cell types in the ground truth.

2.2 Mouse Kidney (MK)

Followed the same simulation procedure, this MK dataset contains the expression of 304 genes by 2,474 spots, annotated into eight distinct cell types. The MK MERFISH dataset (Liu et al. 2023) is available to download from `https://figshare.com/projects/MERFISH_mouse_comparison_study/134213`. We fitted the SpatialCD and STdeconvolve models using the same count matrix with number of topics K as integer values from 2 to 20 and selected $K = 12$, which minimized perplexity and resulted in fewer rare cell types with mean spot proportions below 5%, which are $K = 9$ for SpatialCD and $K = 7$ for STdeconvolve. To ensure a direct comparison with STdeconvolve, and because the SpiceMix model is not able to find the

optimized number of topics K, we fixed $K = 8$, the same as the number of cell types in the ground truth for these two models.

3 Real Data Pre-processing

3.1 Mouse Main Olfactory Bulb (MOB)

We obtained mouse olfactory bulb (MOB) datasets from the original publication (Ståhl et al. 2016), focusing on MOB replicate 8. Data and H&E images of this MOB dataset are available at `https://www.spatialresearch.org/resources-published-datasets/doi-10-1126science-aaf2403/`. Coarse clustering annotations were obtained from STdeconvolve. To ensure a direct comparison with STdeconvolve, we followed the same preprocessing steps. First, we removed genes with fewer than 100 reads detected across spots, and excluded spots with fewer than 100 total gene counts. This filtering process resulted in a cleaned dataset containing 260 spots and 7,365 genes. Next, we selected 255 overdispersed genes using the default generalized additive model (basis = 5) and applied multiple testing to select genes with an adjusted p-value < 0.05. We fit the model using integer values of K from 2 to 20 and selected $K = 12$, which minimized perplexity and resulted in fewer rare cell types with mean spot proportions below 5%.

3.2 Human Pancreatic Ductal Adenocarcinoma (PDAC)

We obtained the PDAC dataset from sample A of the PDAC dataset and are available from the Gene Expression Omnibus (accession number GSE111672) from the original publication (Moncada et al. 2020). To ensure a fair comparison across different deconvolution methods, we standardized the analysis by implementing deconvolution on the same scaled count matrix with 428 spots and 1,379 genes, setting $K = 20$, matching with the number of cell types in the marker gene lists.

4 Comparison with Reference-based Deconvolution under Varying Reference Quality

To further contextualize the performance of SpatialCD relative to supervised approaches, we compared it against RCTD(Cable et al. 2022), a broadly applied reference-based deconvolution method, under three scenarios of varying reference quality using the simulated MPOA dataset. This evaluation follows the simulation framework of STdeconvolve, where ground-truth cell-type proportions and transcriptional profiles are known by construction, enabling direct quantitative comparison via per-spot RMSE.

4.1 Optimal Reference

When the original single-cell MERFISH data was used as an ideal reference, RCTD achieved the lowest overall per-spot RMSE among all methods tested, representing an upper bound for deconvolution performance under idealized conditions (Supplemental Fig S5A). Among the reference-free methods, SpatialCD demonstrated the most competitive performance, consistently outperforming STdeconvolve and SpiceMix in recovering ground-truth cell-type proportions. The deconvolved cell-type proportion maps and Pearson correlation heatmaps confirm that RCTD successfully recovered major cell-type proportions when provided with a well-matched reference (Supplemental Figs S6A-B).

4.2 Missing and Mismatched Reference

In cases where a perfectly matched reference is unavailable, we evaluated RCTD under two challenging conditions: (1) a reference with major neuronal populations (Excitatory and Inhibitory neurons) removed, and (2) an independent scRNA-seq dataset used as a substitute reference. In both cases, RCTD's performance degraded substantially, yielding significantly higher per-spot RMSE compared to both the optimal reference condition and SpatialCD (Supplemental Fig S5B). The deconvolved proportion maps and correlation heatmaps further illustrate this degradation, showing disrupted cell-type assignments and reduced correspondence with ground-truth proportions when neuronal populations were absent from the reference (Supplemental Figs S6C-D). In contrast, SpatialCD's performance remained stable across all conditions, as it infers latent transcriptional programs directly from the spatial transcriptomic data without reliance on an external reference.

4.3 Missing Rare Cell Types

When rare cell types such as ependymal cells were excluded from the reference panel, RCTD erroneously assigned the corresponding spatial spots to transcriptionally similar but distinct populations. This misassignment is clearly visible in the deconvolved proportion maps and correlation heatmaps, where ependymal spots were absorbed into neighboring cell-type categories (Supplemental Figs S6E-F). This reflects a fundamental limitation of reference-based approaches: they are constrained to identify only cell types present in the reference and cannot detect populations absent from the atlas. SpatialCD, being reference-free, recovered these rare transcriptional programs as distinct latent components without being biased by an incomplete reference panel.

In summary, these results demonstrate that while reference-based methods such as RCTD provide strong performance when reference information is complete and precise, their accuracy is highly sensitive to reference quality (Supplemental Figs S5-S6). SpatialCD offers a robust and competitive alternative, particularly in the common practical scenario where a perfectly matched single-cell reference is unavailable or incomplete.

5 Hyperparameter Sensitivity to the Number of Genes

SpatialCD is robust to hyperparameter settings with respect to the number of input genes and does not require additional tuning based on the size of the input gene set. The model operates on a filtered set of overdispersed genes rather than the full gene list, resulting in a compact and informative input matrix regardless of the original dataset scale. For targeted platforms such as MERFISH (e.g., the MPOA dataset with 135 genes), the gene panel is inherently small and is used directly as input. For higher-throughput platforms like Visium, genes are first filtered to a subset of overdispersed genes before model fitting (e.g., 255 genes for the MOB dataset after preprocessing and the top 1,000 overdispersed genes for 10x Visium ST data). In both cases, the same modeling framework and hyperparameter settings can be applied without modification. To ensure a fair comparison across methods, we followed the same preprocessing pipeline as STdeconvolve, so that all methods were applied to identical input matrices.

6 Discrepancy Between Simulation and Real Data Performance

We observed higher quantitative performance in simulated datasets compared to real data, which reflects differences in the evaluation framework rather than limitations of the simulation design. In simulated datasets (MPOA and MK), ground-truth cell-type proportions and gene expression profiles are known by construction, enabling direct evaluation using Pearson's correlation coefficient (PCC) and root mean

squared error (RMSE) against true values. In contrast, real ST datasets lack ground truth. Following prior work (e.g., STdeconvolve (Miller et al. 2022)), we use proxy references derived from anatomical annotations, where spot-level layer labels approximate cell-type proportions and averaged layer-specific expression profiles serve as proxy gene profiles. However, anatomical layers do not correspond to transcriptionally pure cell types and often contain mixtures of cell populations and transitional states, making this evaluation inherently indirect. As a result, lower quantitative agreement in real data is expected. Importantly, despite differences in absolute metrics, the relative performance ranking of methods remains consistent across both simulated and real datasets, supporting the robustness of our conclusions. The observed performance gap reflects differences in evaluation frameworks and the absence of ground truth in real data, rather than unrealistic aspects of the simulation design.

7 Supplementary Figures

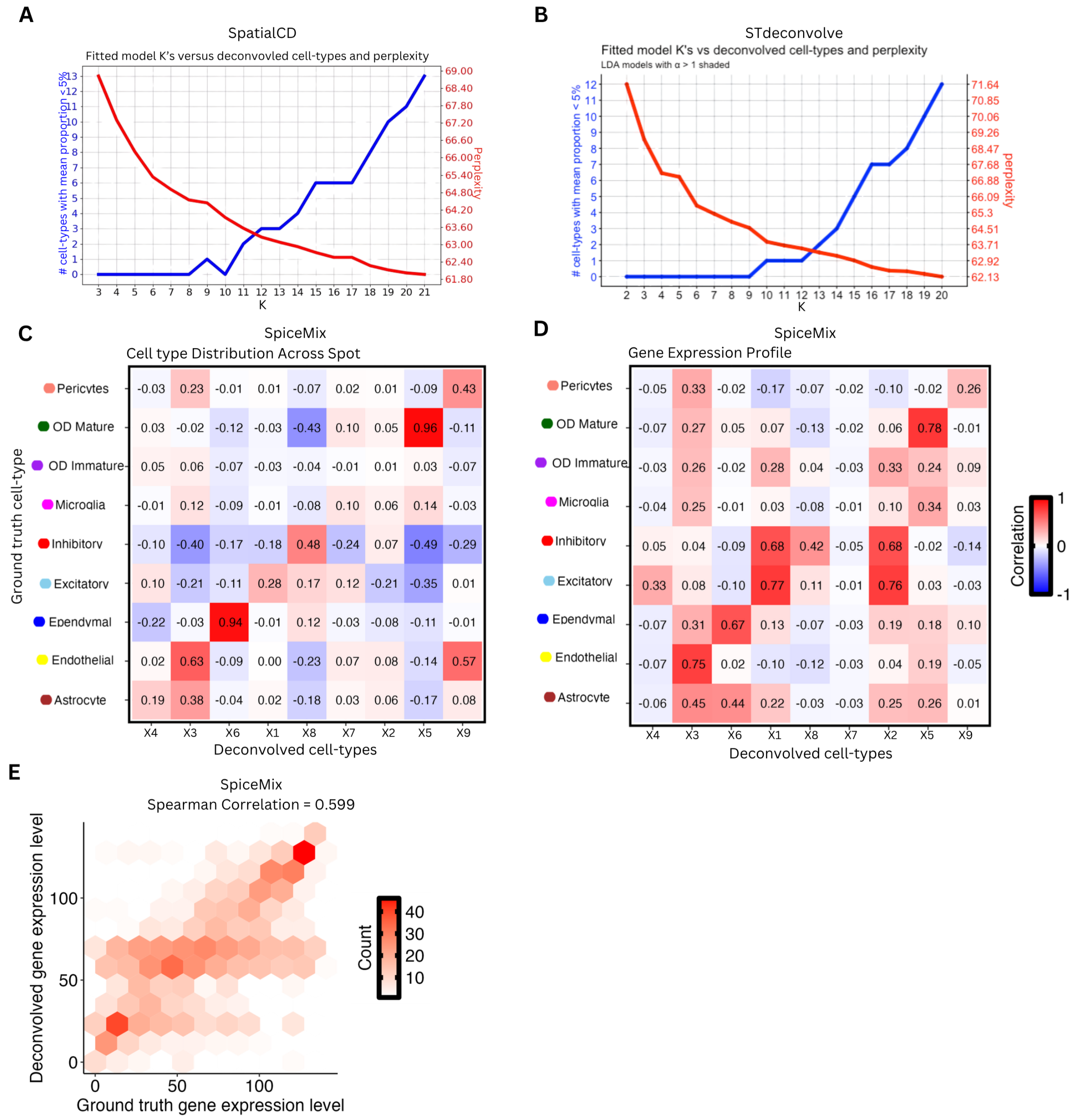

Figure S1: **Deconvolution performance on simulated ST data (MPOA). A.** SpatialCD model selection: perplexity and the number of cell types with mean proportional less than 5% versus different numbers of cell types (K). **B.** STdeconvolve model selection: perplexity and the number of cell types with mean proportional less than 5% versus different numbers of cell types (K). **C.** Pearson's correlation between estimated cell-type proportions (θ) from SpiceMix and the ground truth. **D.** Pearson's correlation between estimated transcriptional profiles (β) from SpiceMix and the ground truth. **E.** Spearman's rank correlation coefficient between gene rankings based on expression levels in deconvolved transcriptional profiles and the corresponding rankings in matched ground truth profiles, using SpiceMix.

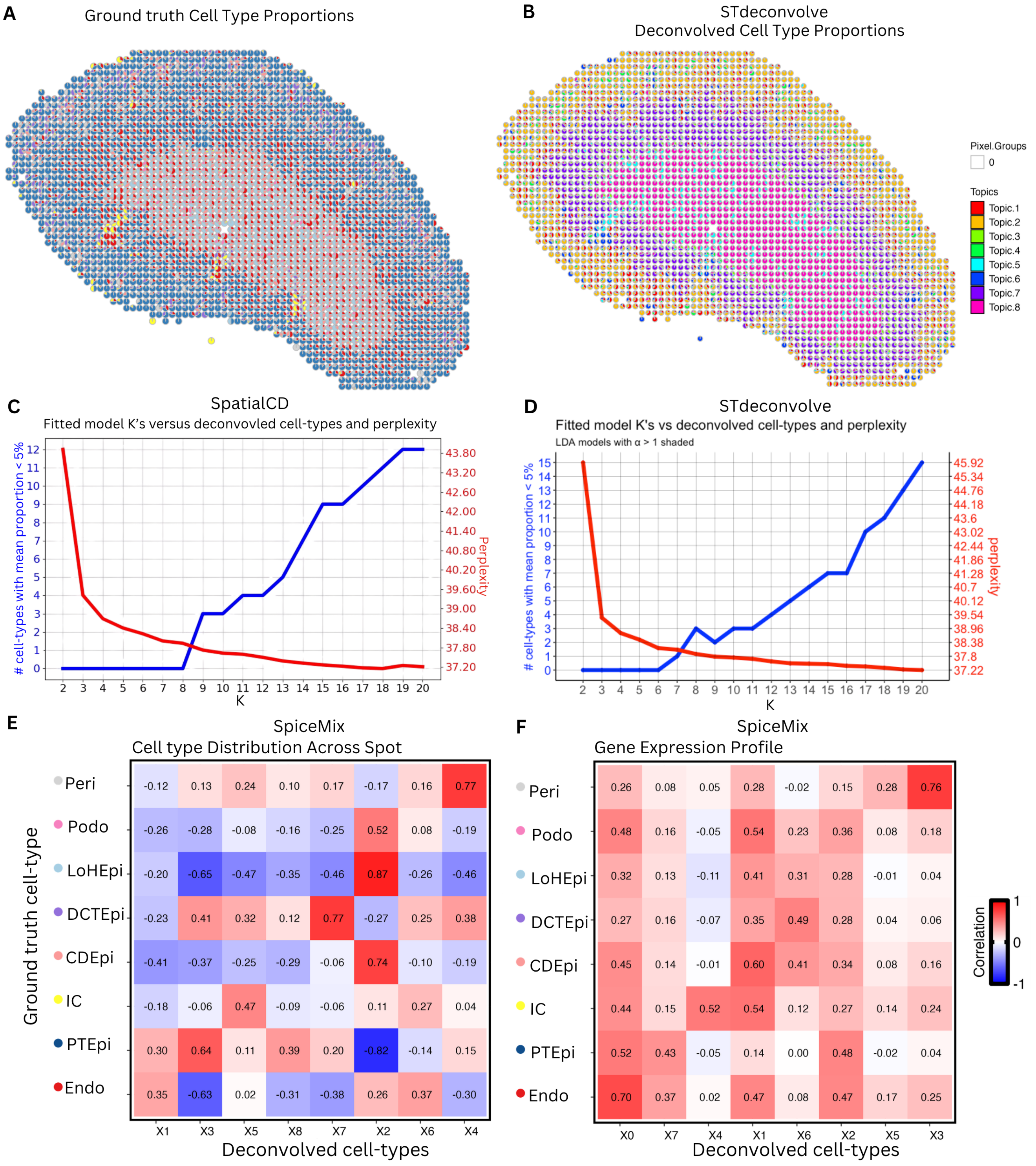

Figure S2: **Deconvolution performance on simulated ST data (MK). A.** Ground truth cell-type proportions represented as pie charts for each simulated spot. **B.** Proportions of deconvolved cell types from STdeconvolve ($K = 8$) represented as pie charts for each simulated spot. **C.** SpatialCD model selection: perplexity and the number of cell types with mean proportion less than 5% versus different numbers of cell types (K). **D.** STdeconvolve model selection: perplexity and the number of cell types with mean proportion less than 5% versus different numbers of cell types (K). **E.** Pearson's correlation between estimated cell-type proportions (θ) from SpiceMix and the ground truth. **F.** Pearson's correlation between estimated transcriptional profiles (β) from SpiceMix and the ground truth.

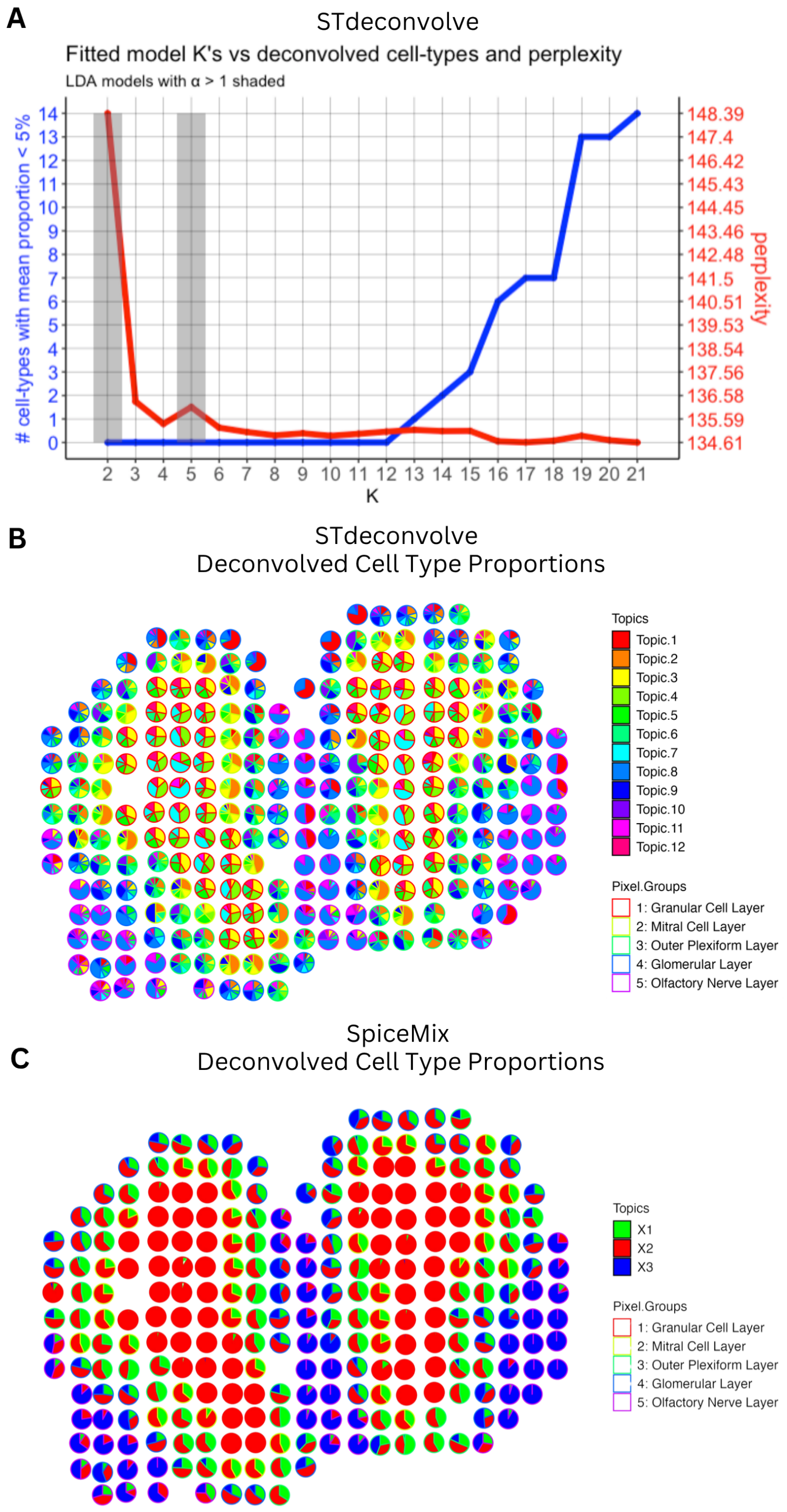

Figure S3: **Deconvolution performance on the MOB ST data. A.** STdeconvolve model selection: perplexity and the number of cell types with mean proportional less than 5% versus different numbers of cell types (K). **B.** Proportions of deconvolved cell types from STdeconvolve ($K = 12$) represented as pie charts for each spot. **C.** Proportions of deconvolved cell types from SpiceMix represented as pie charts for each spot. The output of SpiceMix only shows three cell types with non-zero cell-type compositions.

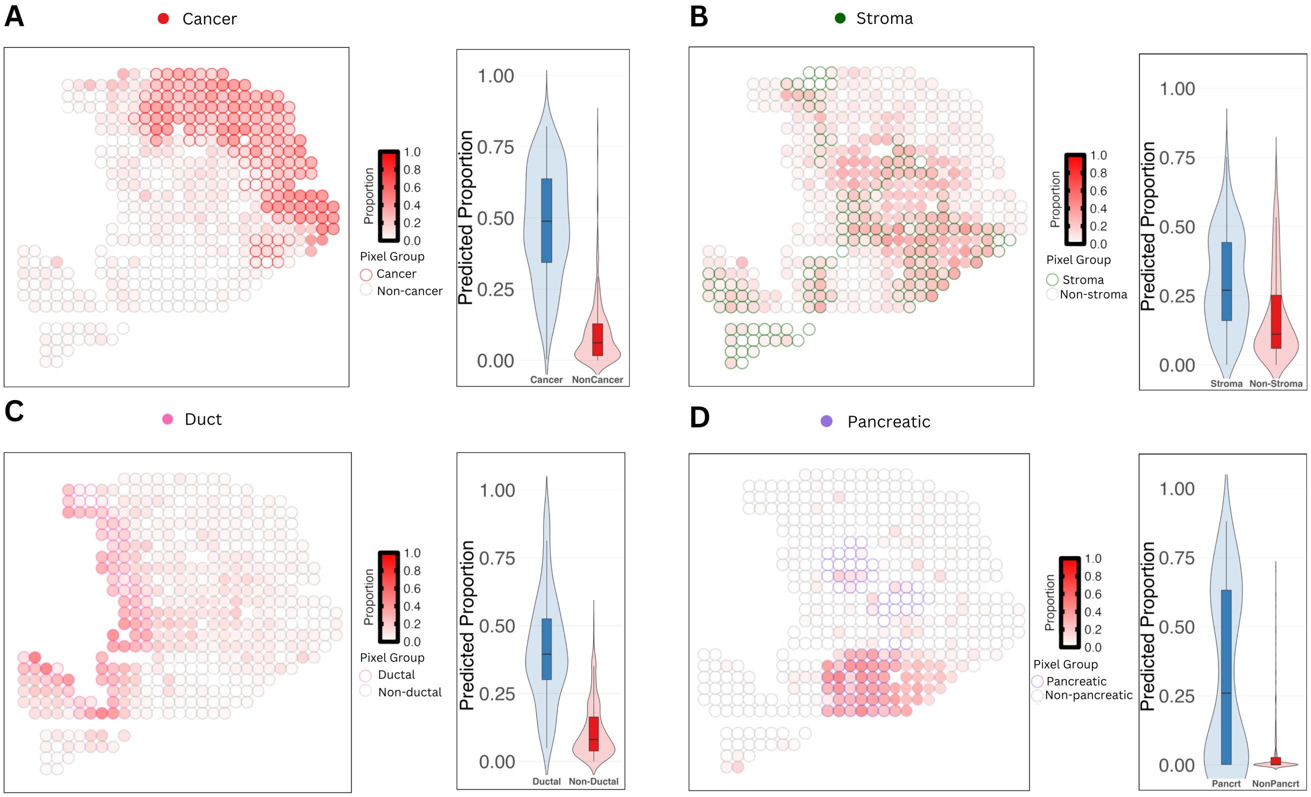

Figure S4: **Deconvolution performance on the PDAC ST data. A.** (Left) Highlights of deconvolved cell types X7 from SpatialCD across annotated cancer spots. (Right) Comparison of cell-type proportions inferred by SpatialCD in cancer regions versus non-cancer regions. **B.** (Left) Highlights of deconvolved cell types X19 from SpatialCD across annotated stroma spots. (Right) Comparison of cell-type proportions inferred by SpatialCD in stroma regions versus non-stroma regions. **C.** (Left) Highlights of deconvolved cell types X15 from SpatialCD across annotated ductal spots. (Right) Comparison of cell-type proportions inferred by SpatialCD in ductal regions versus non-ductal regions. **D.** (Left) Highlights of deconvolved cell types X9 from SpatialCD across annotated pancreatic spots. (Right) Comparison of cell-type proportions inferred by SpatialCD in pancreatic regions versus non-pancreatic regions.

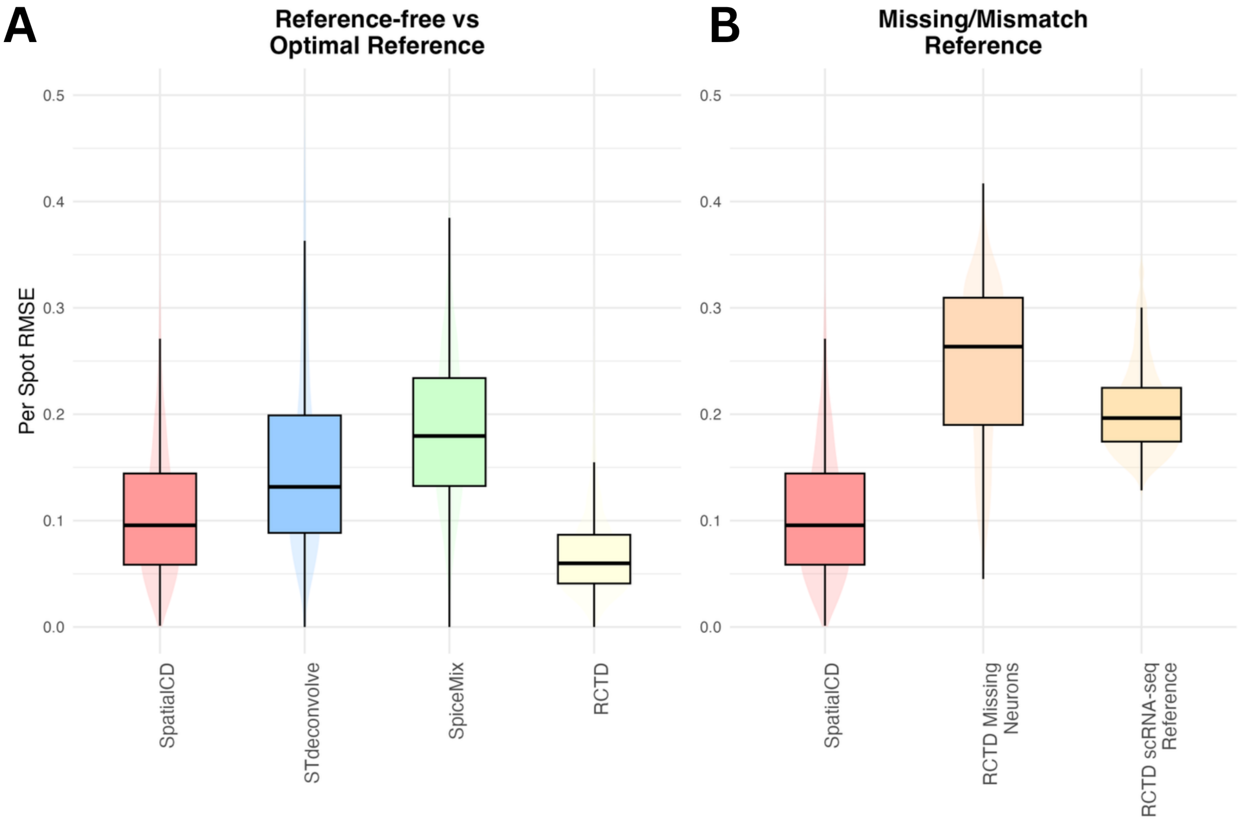

Figure S5: **Comparison of reference-free and reference-based deconvolution methods on simulated MERFISH MPOA spatial transcriptomics data under varying reference conditions.** **A.** Per-spot root mean square error (RMSE) of estimated cell-type proportions relative to ground truth for reference-free methods (SpatialCD, STdeconvolve, SpiceMix) and the reference-based method (RCTD) under optimal reference conditions. **B.** Per-spot RMSE of estimated cell-type proportions under missing and mismatched reference scenarios, comparing the reference-free method (SpatialCD) with the reference-based method (RCTD). Missing reference conditions correspond to the absence of specific cell types (e.g., neurons), while mismatched conditions use an external brain scRNA-seq reference.

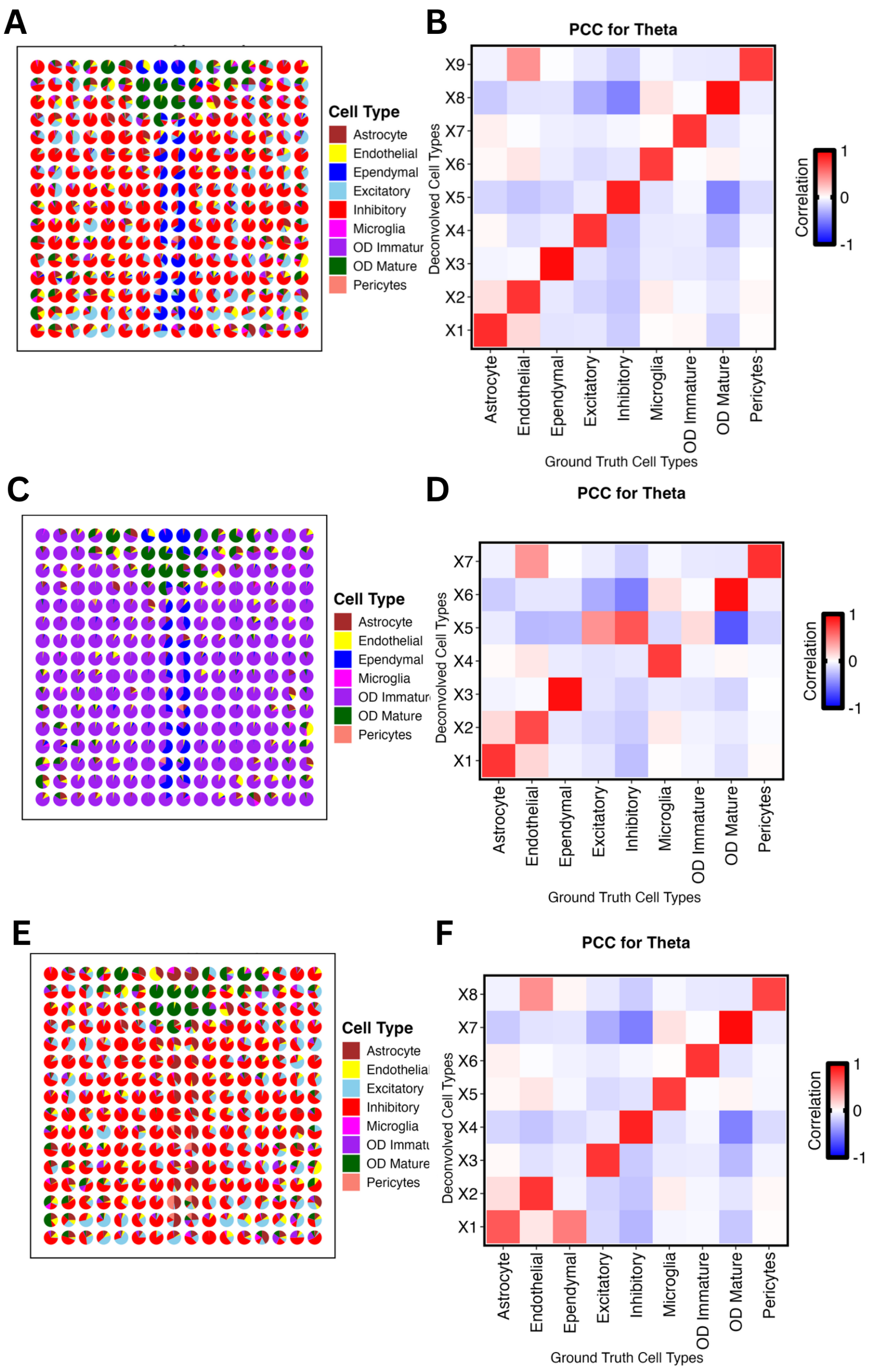

Figure S6: **Cell-type deconvolution results of the reference-based method (RCTD) on simulated MPOA data under varying reference conditions. A.** Deconvolved cell-type proportion distributions under optimal reference conditions. **B.** Pearson's correlation (PCC) between ground truth and estimated cell-type proportions (θ) under optimal reference conditions. **C.** Deconvolved cell-type proportion distributions under missing-reference conditions, where neuronal cell types (Excitatory and Inhibitory) are excluded from the reference. **D.** Pearson's correlation (PCC) between ground truth and estimated cell-type proportions (θ) under missing-neuron conditions. **E.** Deconvolved cell-type proportion distributions under missing rare cell-type conditions (Ependymal cells removed from the reference). **F.** Pearson's correlation (PCC) between ground truth and estimated cell-type proportions (θ) under missing rare cell-type conditions.

References

Blei DM, Ng AY, and Jordan MI. 2003. Latent Dirichlet allocation. *J Mach Learn Res* **3**: 993–1022.

Boyd S, Parikh N, Chu E, Peleato B, and Eckstein J. 2011. Distributed optimization and statistical learning via the alternating direction method of multipliers. *Found Trends Mach Learn* **3**: 1–122.

Boyd S and Vandenberghe L. 2004. *Convex Optimization.* Cambridge University Press, Cambridge.

Cable DM, Murray E, Zou LS, Goeva A, Macosko EZ, Chen F, and Irizarry RA. 2022. Robust decomposition of cell type mixtures in spatial transcriptomics. *Nat Biotechnol* **40**: 517–526.

Liu J, Tran V, Vemuri VNP, Byrne A, Borja M, Kim YJ, Agarwal S, Wang R, Awayan K, Murti A, et al.. 2023. Concordance of merfish spatial transcriptomics with bulk and single-cell rna sequencing. *Life Sci Alliance* **6**: e202201701.

MacKay DJC. 1992. A practical bayesian framework for backpropagation networks. *Neural Comput* **4**: 448–472.

Miller BF, Huang F, Atta L, Sahoo A, and Fan J. 2022. Reference-free cell type deconvolution of multi-cellular pixel-resolution spatially resolved transcriptomics data. *Nat Commun* **13**: 2339.

Moffitt JR, Bambah-Mukku D, Eichhorn SW, Vaughn E, Shekhar K, Perez JD, Rubinstein ND, Hao J, Regev A, Dulac C, et al.. 2018. Molecular, spatial, and functional single-cell profiling of the hypothalamic preoptic region. *Science* **362**: eaau5324.

Moncada R, Barkley D, Wagner F, Chiodin M, Devlin JC, Baron M, Hajdu CH, Simeone DM, and Yanai I. 2020. Integrating microarray-based spatial transcriptomics and single-cell rna-seq reveals tissue architecture in pancreatic ductal adenocarcinomas. *Nat Biotechnol* **38**: 333–342.

Ståhl PL, Salmén F, Vickovic S, Lundmark A, Navarro JF, Magnusson J, Giacomello S, Asp M, Westholm JO, Huss M, et al.. 2016. Visualization and analysis of gene expression in tissue sections by spatial transcriptomics. *Science* **353**: 78–82.

Tansey W and Scott JG. 2015. A fast and flexible algorithm for the graph-fused lasso. *arXiv* p. 1505.06475.

Wolinski P, Charpiat G, and Ollivier Y. 2020. Interpreting a penalty as the influence of a bayesian prior. *arXiv* p. 2001.03601.
